# Changes in Liver Lipidomic Profile in G2019S-*LRRK2* Mouse Model of Parkinson’s Disease

**DOI:** 10.3390/cells12050806

**Published:** 2023-03-04

**Authors:** Yaiza Corral Nieto, Sokhna M. S. Yakhine-Diop, Paula Moreno-Cruz, Laura Manrique García, Amanda Gabrielly Pereira, José A. Morales-García, Mireia Niso-Santano, Rosa A. González-Polo, Elisabet Uribe-Carretero, Sylvère Durand, Maria Chiara Maiuri, Marta Paredes-Barquero, Eva Alegre-Cortés, Saray Canales-Cortés, Adolfo López de Munain, Jordi Pérez-Tur, Ana Pérez-Castillo, Guido Kroemer, José M. Fuentes, José M. Bravo-San Pedro

**Affiliations:** 1 Departamento de Fisiología, Facultad de Medicina, Universidad Complutense de Madrid, 28040 Madrid, Spain; 2 Departamento de Bioquímica y Biología Molecular y Genética, Facultad de Enfermería y Terapia Ocupacional, Universidad de Extremadura, 10003 Cáceres, Spain; 3 Centro de Investigación Biomédica en Red en Enfermedades Neurodegenerativas-Instituto de Salud Carlos III (CIBER-CIBERNED-ISCIII), 28029 Madrid, Spain; 4 Instituto Universitario de Investigación Biosanitaria de Extremadura (INUBE), 10003 Cáceres, Spain; 5 Instituto de Investigaciones Biomédicas “Alberto Sols” (CSIC-UAM), 28029 Madrid, Spain; 6 Departamento de Biología Celular, Facultad de Medicina, Universidad Complutense de Madrid, 28040 Madrid, Spain; 7 Metabolomics and Cell Biology Platforms, Institut Gustave Roussy, 94805 Villejuif, France; 8 Centre de Recherche des Cordeliers, Equipe Labellisée par la Ligue Contre le Cancer, Inserm U1138, Université Paris Cité, Sorbonne Université, 75006 Paris, France; 9 Neuroscience Area of Biodonostia Health Research Institute, Donostia University Hospital, 20014 San Sebastián, Spain; 10 Department of Neurology, Donostia University Hospital, OSAKIDETZA, 20014 San Sebastian, Spain; 11 Ilundain Foundation, 20018 San Sebastian, Spain; 12 Department of Neurosciences, University of the Basque Country UPV-EHU, 20014 San Sebastián, Spain; 13 Instituto de Biomedicina de Valencia-CSIC, Unidad de Genética Molecular, 46010 Valencia, Spain; 14 Unidad Mixta de Genética y Neurología, Instituto de Investigación Sanitaria La Fe, 46026 Valencia, Spain; 15 Institut du Cancer Paris CARPEM, Department of Biology, Hopital Européen Georges Pompidou, AP-HP, 75015 Paris, France

**Keywords:** lipids, liver, LRRK2, metabolome, neurodegeneration, Parkinson

## Abstract

The identification of Parkinson’s disease (PD) biomarkers has become a main goal for the diagnosis of this neurodegenerative disorder. PD has not only been intrinsically related to neurological problems, but also to a series of alterations in peripheral metabolism. The purpose of this study was to identify metabolic changes in the liver in mouse models of PD with the scope of finding new peripheral biomarkers for PD diagnosis. To achieve this goal, we used mass spectrometry technology to determine the complete metabolomic profile of liver and striatal tissue samples from WT mice, 6-hydroxydopamine-treated mice (idiopathic model) and mice affected by the G2019S-*LRRK2* mutation in *LRRK2/PARK8* gene (genetic model). This analysis revealed that the metabolism of carbohydrates, nucleotides and nucleosides was similarly altered in the liver from the two PD mouse models. However, long-chain fatty acids, phosphatidylcholine and other related lipid metabolites were only altered in hepatocytes from G2019S-*LRRK2* mice. In summary, these results reveal specific differences, mainly in lipid metabolism, between idiopathic and genetic PD models in peripheral tissues and open up new possibilities to better understand the etiology of this neurological disorder.

## 1. Introduction

Parkinson’s disease (PD) is the second most common neurodegenerative disorder [1], only below Alzheimer’s disease. In recent years, it has experienced a very rapid growth in prevalence, becoming one of the main causes of disability worldwide [2]. PD is a progressive neurological disorder mainly characterized by the loss of dopaminergic neurons in the substantia nigra pars compacta (SNpc), a critical area for movement control in the brain [3], leading to the major clinical motor symptoms of the disease, such as bradykinesia and rigidity [4,5]. Thus, there is, on the one hand, a direct and evident relationship between disorders at the neural level and PD, but on the other hand, it is known that this disease is related to peripheral organs, including the liver. In fact, alpha synuclein accumulations have been observed both in brain [6] and liver [7]. Moreover, some common urinary markers, as 8-hydroxy-2′-deoxyguanosin, have been identified for PD [8] and chronic liver disease patients [9] and a correlation between patients with cirrhosis and PD has also been hypothesized and verified by a significant improvement of motor symptoms after the first year of liver transplantation in patients with cirrhosis and PD [10].

The bases of the etiology of this disease have not been fully deciphered, so exposure to environmental toxins, genetic factors and aging are currently accepted as the major triggers of this type of neurodegeneration [11]. Most patients diagnosed with PD (approximately 80–85%) have primary parkinsonism or idiopathic PD; that is, the cause of the disease remains unknown. Exposure to the catecholaminergic neurotoxin 6-OHDA has been widely used to generate PD models, which are considered appropriate for studying the idiopathic form of PD [12]. Nevertheless, a small percentage of patients manifest a genetic form of PD. There are multiple PD-related genes (called PARK genes) unequivocally linked to the inherited monogenic disease. Mutations in *SNCA* (*PARK1/4*), *Parkin* (*PARK2*), *PINK1* (*PARK6*), *DJ-1* (*PARK7*), *LRRK2* (*PARK8*) and *ATP13A2* (*PARK9*) are responsible for the autosomal dominant or recessive mode of inheritance for PD. There are few missense variants of *LRRK2*/*PARK8* that have been confirmed to increase the risk of PD, including variants G2019S, N1437H, R1441C/G/H/S, Y1699C, and I2020T. However, the substitution of the serine residue by a glycine residue in exon 41 of the protein kinase domain in *LRRK2* (G2019S mutation) is the most common mutation across 51 countries [13], accounting for 1% of sporadic PD cases and 4% of familial PD cases, among all cases [14,15]. It is well described that the presence of the G2019S mutation induces an increase in the kinase activity of this protein, which is the precursor of neuronal damage associated with the disease [16]. In fact, inhibitors of LRRK2 kinase activity are one of the best potential therapeutics for the disease caused by the said mutation [17]. The exact molecular mechanisms underlying LRRK2-associated PD pathology are far from clear; however, it is known that alterations in this gene affect important cellular processes such as microtubule dynamics, vesicular trafficking, synaptic transmission or autophagy [18,19,20,21].

Omics analysis, especially metabolomics, is a very comprehensive tool for identifying molecular networks related to the pathogenesis of this little-known disease [22]. There are multiple metabolomic studies based on the analysis of metabolites in cerebrospinal fluid [23], blood samples [24], urine [25], feces [26] or neuronal tissue [27]. However, analysis of liver cells has not been performed in neurodegenerative models of this disease. Our aim was to study the metabolic alterations produced in the liver and SNpc tissues samples after developing genetic or acute intoxication PD models. This was accomplished by examining the complete metabolomic profile of hepatocyte and striatum cells in both mouse models.

## 2. Materials and Methods

Mass spectrometry was used to perform a metabolomic analysis of striatum (*n* = 5 mice/group) and liver (*n* = 4–5 mice/group) tissues in three different mouse models of PD and respective control mice: i, control mice (non-parkinsonian WT mice, untreated); ii, GS-PD mice (parkinsonian mice with whole body G2019S-*LRRK2* mutation); iii, ai-PD mice (parkinsonian WT mice, treated with neurotoxin 6-OHDA).

Mouse strains and housing: All animal experiments were allowed by the “Ethics Committee for Animal Experimentation” of the Biomedical Research Institute “Alberto Sols” (CSIC-UAM) in Madrid (Spain) and performed in accordance with the European Communities Council Directive (2010/63/EEC) and national regulations (normative RD1386/2018). Adult male WT-*LRRK2* and G2019S-*LRRK2* transgenic mice (Tg) (3 months old, 25–30 g) were obtained from Jackson Laboratories and backcrossed in house. Genotyping was performed via PCR using the following oligonucleotide primers: 5′-ATTACCATGGTTCGAGGTGA-3′ (forward) and 5′-CAAGTGTCTGCAGGAAGGTT-3′ (reverse) for G2019S-*LRRK2*; 5′-CTAGGCCACAGAATTGAAAGATCT-3′ (forward) and 5′-GTAGGTGAAATTCTAGCATCATCC-3′ (reverse) for an internal-positive control. About two to three animals were housed per cage with free access to chow and liquid under a 12 h light/dark cycle. Special care was taken to minimize pain and discomfort in animals.

Acute intoxication PD model: This model was induced as previously described [28]. Using a stereotaxic apparatus (Kopf Instruments, Tujunga, CA, USA), 6-OHDA (5 μg in 2.5 μL of saline with 0.02% ascorbic acid) was unilaterally injected into the SNpc of anesthetized mice at the subsequent coordinates from bregma: posterior, −3.2 mm; lateral, +2.0 mm; and ventral, +4.7 mm, with the skull flat between lambda and bregma, according to this mouse brain atlas [29]. The mice were then housed for quick recovery. Mice were sacrificed 45 days after the 6-OHDA-induced damage (i.e., at 4.5 months of age), at which time the loss of dopaminergic neurons was over 65%, in line with what was described in human patients at the time of diagnosis of the disease. In both murine and human, it is the intermediate (mid-final) stage of neurodegeneration. In addition to motor alterations detected in animals subjected to the apomorphine injection test, we have previously demonstrated through histological and immunohistochemical analysis an increase in the production of proinflammatory factors (activation of microglia) and dopaminergic death in the substantia nigra [28].

Tissue sample preparation for metabolomic analyses: About 30 mg of samples for each condition were solubilized into 1.5 mL tubes with ceramic beads with 1 mL of cold lysate buffer consisted of ISTD (MeOH/Water, 9/1, −20 °C). They were then homogenized three times for 20 s at 5500 rpm using Precellys 24 tissue homogenizer (Bertin Technologies, Montigny-le-Bretonneux, France), and centrifuged for 10 min at 15,000× *g*, 4 °C. Subsequently, the upper phase of supernatant was divided into two volumes of 300 µL, one was used for gas chromatography coupled by mass spectrometry (GC/MS) experiment in microtube, and the other was used for ultrahigh pressure liquid chromatography coupled by mass spectrometry (UHPLC/MS) experimentation.

Regarding GC-MS aliquots, the volume of 300 µL was transferred to glass tubes and evaporated. Subsequently, we added 50 µL of methoxyamine (20 mg/mL in pyridine) to the dry extracts and stored the samples overnight at room temperature in the dark. The next day, we added 80 µL of MSTFA and the final derivatization occurred at 40 °C for 30 min. Afterwards, samples were transferred in vials and directly injected into GC-MS.

Concerning the UHPLC-MS aliquots, the volume of 300 µL was dried in microtubes at 40 °C in a pneumatically assisted concentrator (Techne DB3, Staffordshire, UK). The dry extracts were dissolved with 200 µL of MilliQ water. Samples were transferred in LC vials and injected into UHPLC-MS or stored at −80 °C until injection.

Targeted analysis by GC coupled to triple quadrupole (QQQ) mass spectrometry: GC-MS/MS acquisitions were performed on a 7890B gas chromatograph coupled to a triple quadrupole 7000C detector (both from Agilent Technologies, Santa Clara, CA, USA), equipped with an electronic impact source (EIS) operating in positive mode and a 30 m × 0.25 mm I.D. × 0.25 mm film thickness HP5MS capillary column (Agilent Technologies). Sample aliquots of 1 µL were inoculated into an inlet operating in splitless mode and set at 250 °C. Helium gas flow rate was fixed at 1 mL/min and the septum purge flow at 3 mL/min. The temperature was programmed as follows: 60 °C for 1 min, +10 °C/min up to 210 °C, hold for 3 min, +5 °C/min up to 325 °C and hold for 5 min. The transfer line and ion-source temperatures were 250 °C and 230 °C, respectively.

Targeted analysis by UHPLC coupled to triple quadrupole (QQQ) mass spectrometry: Targeted analysis was conducted on a RRLC 1260 system coupled to a triple quadrupole 6410 detector (Agilent Technologies), armed with an electrospray source operating in positive mode. Gas temperature was set at 350 °C, gas flow at 12 L/min, and capillary voltage at 3.5 kV. Sample aliquots of 10 µL were injected on a Zorbax Eclipse XDB-C18 column (100 mm × 2.1 mm, particle size 1.8 mm, Agilent Technologies), protected by an XDB-C18 guard column (5 mm × 2.1 mm, particle size 1.8 mm) and heated at 40 °C. The gradient mobile phase consisted of 2 mM of dibutyl ammonium acetate (DBAA) in water (A) and acetonitrile (B). The flow rate was set at 0.2 mL/min, and the gradient modified as follows: initial condition (90% phase A and 10% phase B) was maintained for 4 min, from 10% to 95% phase B over 3 min. The column was washed using 95% mobile phase B for 3 min and equilibrated using 10% phase B for 3 min. The autosampler was kept at 4 °C.

Pseudo-targeted analysis of intracellular metabolites by UHPLC combined to a Q-Exactive mass spectrometer. Reversed phase acetonitrile method: The profiling experiment was done with a Dionex Ultimate 3000 UHPLC system (Thermo Scientific, Waltham, MA, USA) coupled to a Q-Exactive (Thermo Scientific) equipped with an electrospray source operating in both positive and negative mode and full scan mode from 100 to 1200 *m*/*z*. The Q-Exactive parameters were: sheath gas flow rate 55 au, auxiliary gas flow rate 15 au, spray voltage 3.3 kV, capillary temperature 300 °C, S-Lens RF level 55 V. The mass spectrometer was calibrated with sodium acetate solution specific for low mass calibration.

Samples (10 μL) were injected on an SB-Aq column (100 mm × 2.1 mm particle size 1.8 μm) from Agilent Technologies, protected by a guard column XDB-C18 (5 mm × 2.1 mm particle size 1.8 μm) and warmed at 40 °C by a pelletier oven. The gradient mobile phase consists of 0.2% of acetic acid (A) and acetonitrile (B) in water. The flow rate was set to 0.3 mL/min. Initial condition was 98% phase A and 2% phase B. Molecules were then eluted using a gradient from 2% to 95% phase B in 22 min. The column was cleaned using 95% mobile phase B for 2 min and equilibrated using 2% mobile phase B for 4 min. The autosampler was kept at 4 °C. Peak detection and integration were carried out using the Thermo Xcalibur quantitative software (2.1.)

Quantification and statistical analysis: The data are reported as the means ± standard error of the mean (SEM). The number of independent data points (N) is indicated in Table S1. For statistical analyses, *p*-values were estimated by one-way ANOVA (analyzing metabolites individually), or Pearson’s correlation coefficients with 95% confidence intervals (Pearson’s correlation coefficient (R)). Differences were considered statistically significant when *p*-values were: ° (*p* < 0.1), * (*p* < 0.05), ** (*p* < 0.01), *** (*p* < 0.001) and **** (*p* < 0.0001).

## 3. Results

### 3.1. Metabolic Changes Observed in Nerve Cells of Genetic and Acute Intoxication Models of PD

A metabolomic approach was performed to study potential markers involved in the development of parkinsonian mice. For this, nervous and hepatic tissues extracted from mice with genetic model (GS-PD, due to G2019S-*LRRK2* mutation) or acute intoxication model (ai-PD, WT mice treated with 6-OHDA) were analyzed to observe the complete metabolomic profile in striatum (Table S1) and hepatocyte cells (Table S2).

**Figure 1:**
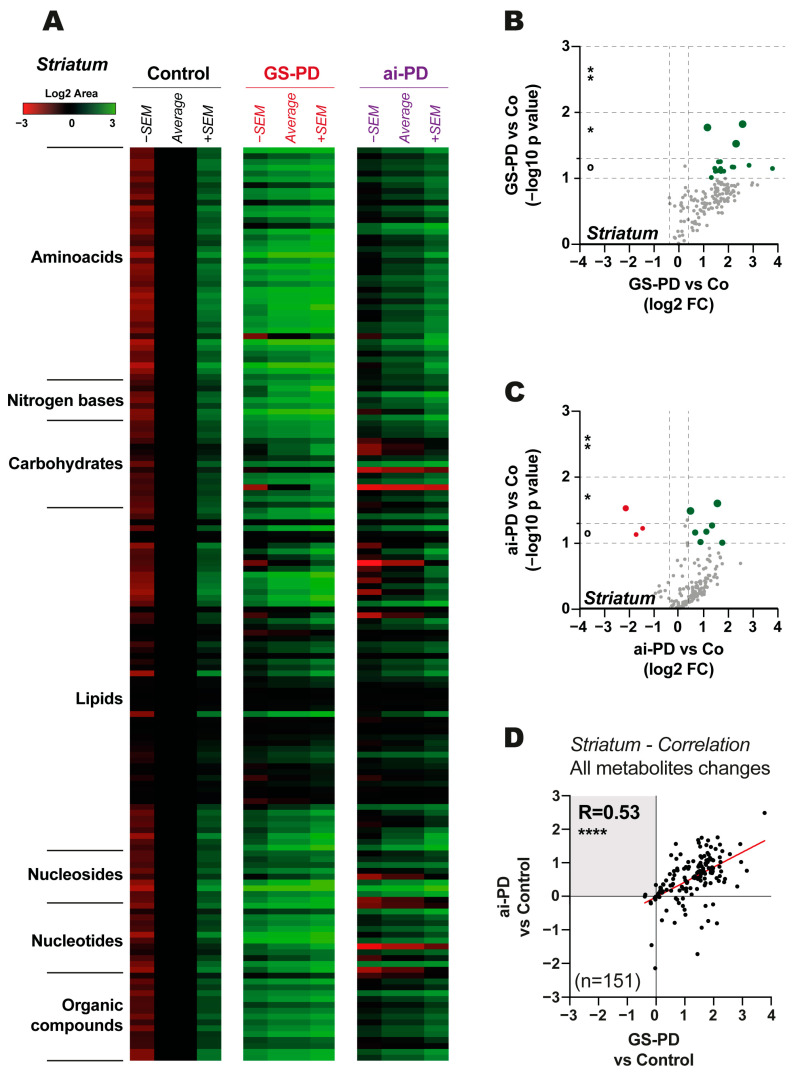
Results of metabolic changes observed in striatum tissues for the control (Co) and parkinsonian genetic (GS-PD) or due to acute intoxication (ai-PD) groups (*n* = 5). (**A**) Heatmap with the average of the log2 area (± standard error of the mean (SEM)) showed by metabolite groups (amino acids, nitrogen bases, carbohydrates, lipids, nucleosides, nucleotides, and organic compounds). (**B**,**C**) Volcano plot graphs are shown. The log2 FC shows changes observed on GS-PD model (**B**) or ai-PD model (**C**) by comparison to the control mice for each metabolite (represented by each dot). The −log10 *p* value represents non-significant (grey color) or significant (red represents significantly down-regulated metabolites, whereas green represents significantly up-regulated metabolites. (**D**) Correlation analysis between changes observed in striatal neurons of GS-PD compared to controls and ai-PD compared to controls. Statistical analysis was performed by obtaining *p* value (° (*p* < 0.1), * (*p* < 0.05), ** (*p* < 0.01), **** (*p* < 0.0001)), and Pearson’s correlation coefficient (R) between the noted changes.

Regarding the metabolomic results obtained from the nerve cells, we noticed an overall increase in most metabolites in the GS-PD model (Figure 1A,B) and in the ai-PD model (Figure 1A,C). When we analyzed all metabolite changes observed in the striatum, we found a good correlation (Pearson correlation coefficient (R = 0.53 and *p* value < 0.0001) (Figure 1D). These results indicate that metabolic modulations are independent of the etymological origin of the disease.

Performing an in-depth analysis to independently study what is happening in the different metabolic pathways, we can determine which pathways are more or less altered in the two PD models studied (Figure S1). Thereby, we have observed a very strong correlation in most routes, with a great significance (amino acids: R = 0.53 and *p* value < 0.001; organic compounds; R = 0.88 and *p* value < 0.0001; lipids R = 0.58 and *p* value < 0.0001; nucleosides: R = 0.79 and *p* value = 0.02; carbohydrates: R = 0.87 and *p* value < 0.0001) (Figure S1A–E). It should be noted that in the carbohydrates pathway, maltose and sucrose were exclusively reduced in ai-PD (Figure S1E).

We also found different modulations between both PD models when we focused on nucleotides (R = −0.003, *p* value = 0.99, Figure S1F) and nitrogen bases (R = −0.57 and *p* value = 0.18, Figure S1G). By looking into the different metabolites regulated in these pathways, we observed that NADH was increased in GS-PD, but decreased in ai-PD models (Figure S1F). Moreover, uric acid levels were exclusively raised in GS-PD mice (Figure S1G).

### 3.2. Metabolic Changes Observed in the Hepatocytes of Genetic and Acute Intoxication Models of PD

**Figure 2:**
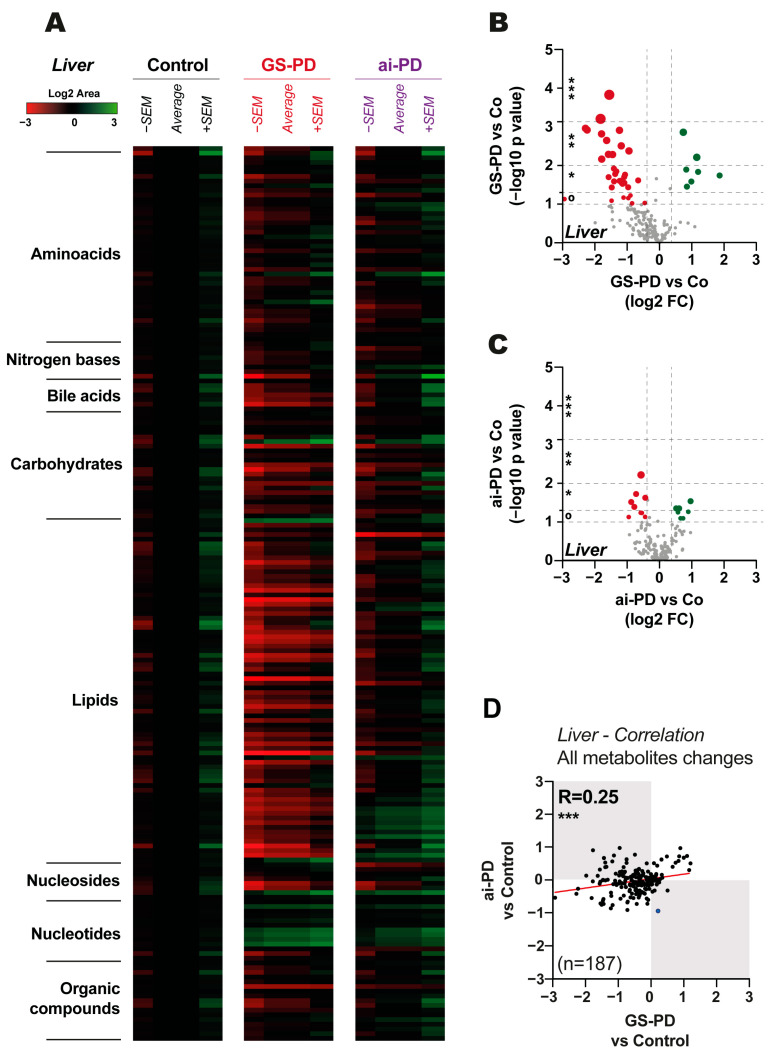
Results of metabolic changes observed in hepatic tissues for the control (Co) and parkinsonian genetic (GS-PD) or due to acute intoxication (ai-PD) groups (*n* = 4–5). (**A**) Heatmap with the average of the log2 area (± standard error of the mean (SEM)) showed by metabolite groups (amino acids, nitrogenous bases, carbohydrates, lipids, nucleosides, nucleotides and organic compounds). (**B**,**C**) Volcano plot graphs are shown. The log2 FC indicates the changes observed on GS-PD model (**B**) or ai-PD model (**C**) in comparison to control mice for each metabolite (represented by each dot). The −log10 *p* value represents non-significant (grey color) or significant (red represents significantly down-regulated metabolites, whereas green represents significantly up-regulated metabolites. (**D**) Correlation analysis between changes observed in striatal neurons of GS-PD or ai-PD compared to controls. Statistical analysis was performed by obtaining *p* value (° (*p* < 0.1), * (*p* < 0.05), ** (*p* < 0.01), *** *p* < 0.001), and Pearson’s correlation coefficients (R) between the observed changes).

Regarding the results obtained in liver cells, we found a drop in numerous metabolites, which occurs exclusively in the genetic model (Figure 2A,B), with no significant changes in the liver of ai-PD model (Figure 2A,C). Indeed, analyzing different metabolic responses of the two PD induction models in the liver, the correlation between the whole metabolite changes observed in both models is very low (R = 0.25) (Figure 2D), corroborating the presence of numerous modulations of hepatic metabolism exclusively in the GS-PD model but not in the acute intoxication model.

To better understand which metabolites are exclusively modulated in GS-PD or ai-PD mice, we performed a correlation analysis between the modulations observed in parkinsonian mice from both models. We notified a similar response in the parkinsonian mice from both models in some pathways: amino acids: R = 0.35 and *p* value = 0.02; carbohydrates: R = 0.83 and *p* value < 0.0001; organic compounds: R = 0.66 and *p* value = 0.002; nucleotides: R = 0.83 and *p* value = 0.0005; nucleosides: R = 0.62 and *p* value = 0.09 (Figure S2A–E).

However, we found that nitrogen bases (GS-PD vs. ai-PD correlation: R = 0.57 and *p* value = 0.14), bile acids (GS-PD vs. ai-PD correlation: R = 0.13 and *p* value = 0.79) and lipids (GS-PD vs. ai-PD correlation: R = −0.0001 and *p* value = 0.99) are mainly modulated in the genetic model, but not in the acute intoxication model in hepatocytes. (Figure S2F–H).

### 3.3. Changes in Lipid Metabolites in Genetic or Acute Intoxication PD Models Liver

**Figure 3:**
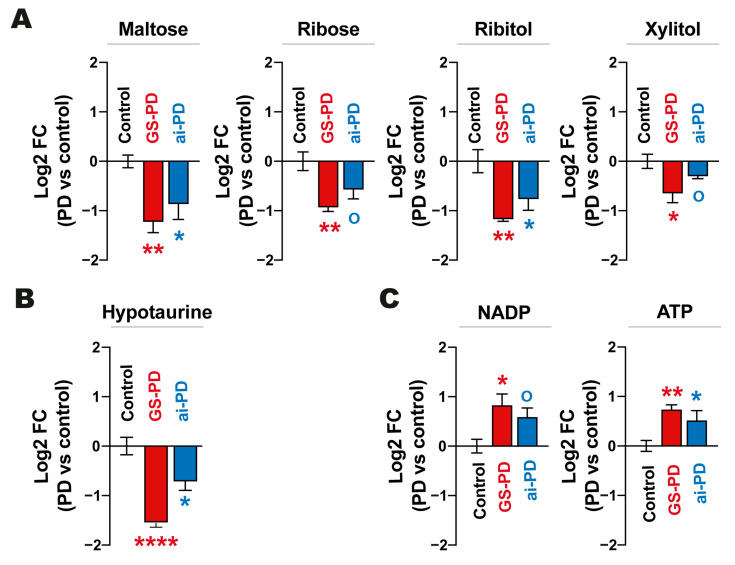
Histograms showing the average (± standard error of the mean (SEM)) of log2-fold change (Log2 FC) concentrations of different metabolites significantly decreased (**A**,**B**) or increased (**C**) in the liver of genetic (carrying the p.G2019S mutation in *LRRK2*; GS-PD) and acute intoxication PD (ai-PD) mouse models (*n* = 4–5). For statistical analyses, *p*-values were calculated by one-way ANOVA (analyzing the metabolites individually) and differences were evaluated as statistically significant when *p*-values were: ° (*p* < 0.1), * (*p* < 0.05) and ** (*p* < 0.01).

According to the results obtained in the liver extract, genetic models and those resulting from the acute intoxication of PD exert a similar metabolic modulation. Within the metabolic routes in which we observed a similar behavior in both models, we can highlight a decrease in maltose disaccharide carbohydrates such as the pentoses ribose, ribitol and xylitol (Figure 3A), as well a drop in hypotaurine levels (Figure 3B). Finally, we found an increase in the NADP and ATP levels in parkinsonian mice liver, regardless of the origin of the disease (Figure 3C).

**Figure 4:**
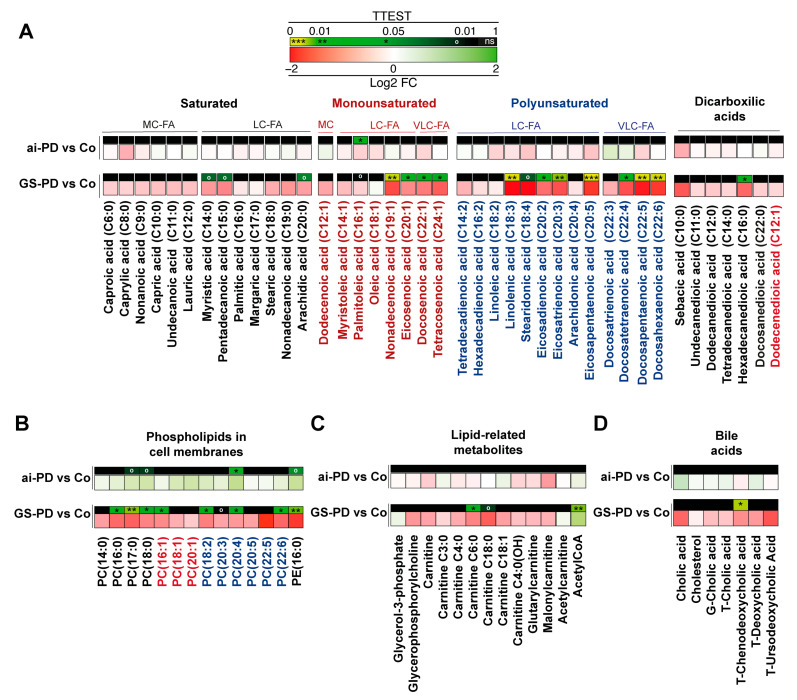
Heatmaps showing the TTEST (*p* value) on the square above, and log 2-fold change (Log2 FC) on the square below for the different concentration of fatty acids (FA) (**A**), phospholipids in cell membranes (**B**) lipid-related metabolites (**C**) and bile acid metabolites (**D**) in the liver of WT group and genetic (carrying the p.G2019S mutation in *LRRK2*; GS-PD) and acute intoxication PD (ai-PD) mouse models (*n* = 4–5). For statistical data, *p*-values were estimated by one-way ANOVA (analyzing the metabolites separately) and differences were considered significant when *p*-values were: ° (*p* < 0.1), * (*p* < 0.05), ** (*p* < 0.01) and *** (*p* < 0.001). G, glycine; LC, long-chain; MC, medium-chain; PC, phosphatidylcholine; PE, phosphatidylethanolamine; T, taurine; VLC, very long-chain.

As previously mentioned, genetic and acute intoxication PD models exert similar metabolic modulation in the liver, with the exception of lipid metabolism, which is highly modulated in the genetic model (Figure S2H). Analyzing the lipid metabolism in this organ in depth, we observed an important decrease in fatty acids (FAs), in the genetic GS-PD model with respect to healthy mice; such as long-chain saturated FAs (C14:0, C15:0 and C20:0), monounsaturated long- and very long-chain FAs (C19:1, C20:1, C22:1, C24:1) and polyunsaturated long- and very long-chain (C18:3, C18:4, C20;2, C20;3, C20;5, C22;4, C22;5, C22;6). However, no significant variations were observed for dicarboxylic acids (except for hexadecanedioic acid, C16:0) (Figure 4A). Additionally, there were several changes in cell membrane phospholipids, with an important decrease in phosphatidylcholine (PC) (PC 16:0, PC 17:0, PC 18:0, PC 16:1, PC 18:2, PC 20:3, PC 20:4, PC 22:6) and phosphatidylethanolamine (PE 16:0) in parkinsonian GS-PD mice liver (Figure 4B). Finally, we noticed a decrease for general carnitines, specifically carnitines C6:0 or C18:0, and an increase in acetyl-CoA levels on this genetic model (Figure 4C). Regarding bile salts, there was a general decrease in hepatic levels of all intermediate metabolites considered exclusive in mice carrying the G2019S-*LRRK2* mutation (Figure 4D).

## 4. Discussion

The factors that contribute to the onset of PD are highly variable. Indeed, the loss of dopaminergic terminals and lack of dopamine release have been reported in the striatum from models as varied as PD patients, transgenic animal models of PD, or different toxin-induced models [5,30,31,32]. Understanding the differences between these disease triggers is very important to improve our comprehension and to generate specific therapies for each PD risk factor. For this, it would be interesting to detect common metabolic or specific modulations between all disease triggers. It is also essential to assess whether these factors have a metabolic influence, not only on the nervous system, but on the periphery as well. Metabolic problems associated with PD are not just restricted to plasma and nervous tissue but also to *PARK* gene defects and liver damage, such as the parkin defect and its relationship with alcohol-induced liver injury and steatosis in mice [33]. The present study showed a metabolomic evaluation of brain and liver tissues in two different acute intoxication and genetic PD models.

Analyzing the metabolic profiles obtained from the striatum, we observed that most metabolic changes occur in parallel in all parkinsonian mice, regardless of the genetic origin or due to acute intoxication of the disease. In general, an increase in a wide range of amino acids, organic compounds, lipids or nucleosides was reported (Figure 1 and Figure S1A–D). It is curious that in the acute intoxication model, but not in the genetic model, a decrease in sucrose and maltose levels is observed, and some previous studies have linked PD to problems with carbohydrate metabolism [34]. However, analyzing all the data, we have not been able to observe significant modulations of the main metabolites related to carbohydrate metabolism, such as glucose, glucose-6-phosphate or fructose-6-phosphate in the striatum (Table S1) or in the liver (Table S2), so there do not appear to be important changes in the carbohydrate pathways in the striatum in both models. Therefore, we can conclude that the damage produced by the G2019S-*LRRK2* mutation throughout the body, and by acute 6-OHDA intoxication, are very similar in terms of metabolic changes in the striatum of diseased mice compared to healthy mice.

Nevertheless, when we check the metabolic profiles in the liver of GS-PD and ai-PD mice, the number of differences between both models is much greater (Figure 2). Analyzing in depth these differently modulated metabolites, we observed that almost all of them are part of lipid metabolism pathways (Figure S2). The lipidomic approaches in already published articles highlighted important functions of lipids and, in particular, dysfunctions in lipid metabolism in the pathogenesis of protein misfolding diseases, including PD [35,36]. Interestingly, in the last decade, there has been a growth in the study of the interaction between macroautophagy and lipid metabolism [37,38,39] and prior to this study, we have shown an excess in autophagy flux in fibroblasts from patients with the G2019S-*LRRK2* mutation [20,40]. Moreover, despite the fact that autophagy can help to eliminate hepatic steatosis [41], and that it is a powerful tool in multiple diseases [42], including neurodegenerative and liver-associated diseases, an excess of this autophagy response could be equally negative [20,40]. Thus, it would be very interesting to study the specific role that the aberrant autophagy associated with the GS-PD mice has in the deregulation of lipid metabolism in the liver tissue samples.

Going in depth into the observed changes, we noticed a decrease in mono- and polyunsaturated LCFA and VLCFA levels exclusively in the liver of GS-PD mice, but the level of these metabolites was not modified in the acute intoxication model (Figure 4), as previously described in rats treated with 6-OHDA [43]. Therefore, it seems that there is a possible implication of the LRRK2 protein in the hepatic control of lipids. In this sense, previous studies have shown that activity of fatty acid oxidation is increased in *LRRK2*-overexpressing liver hepatic carcinoma cells [44]; conversely, in hepatocytes and stellate cells of *LRRK2*-KO mice, the lipid droplets accumulate more than in *LRRK2*-WT animals [45,46]. Indeed, the present results indicate that there is an increase in the oxidation of long- and very long-chain fatty acids in the genetic model, after having observed a decrease in fatty acids and a consequent increase in the production of NADH, NADPH, acetyl CoA and ATP (Figure 3 and Figure 4C). Importantly, this increase in acetyl-CoA appears to be consistent with the increased levels of acetylated proteins observed in patients with G2019S-*LRRK2* mutation [47,48]; this phenotype was not observed in idiopathic PD patients.

In addition, and also related to lipid metabolism, we found that PC and PE, major components of biological membranes, are exclusively decreased in hepatocytes obtained from GS-PD mice (Figure 4B and Figure S3). Interestingly, a decrease in PC has already been observed in other samples, as plasma and frontal cortex from PD patients [49], in brain from animal PD models and in goldfish models treated with MPTP [49,50]. It must be highlighted that G2019S-*LRRK2* mutation has been directly linked with α-synucleinopathies [51] and that the α-Syn deposits are not confined to the organs of the central nervous system and are found in other organs and cells, including hepatocytes of different animal and cellular models of PD and in humans [7]. α-Syn is a protein that localizes to curved and ordered membranes inside the cell, and changes in PC concentrations in this membrane can affect α-syn fluidity and conformation, leading to its aggregation [52]. Considering that modulations in the physiological fluidity of the membrane may promote the accumulation of insoluble materials associated with PD [53], and that the liver is involved in the clearance of pathological protein aggregates [7], it can be hypothesized that this decrease in PC and PE in the liver could have a negative effect on the role played by this organ in the cleaning of α-syn pools.

Finally, there is a relationship between ceramides and sphingolipids and PD [54,55]. Defective sphingolipid pathways are reported through some clinical studies on PD subclasses [55], and *LRRK2* KO mice showed elevated levels of ceramides in the brain [56]. In addition, treatment with specific LRRK2 inhibitors (in assays of clinical phase) increased β-Glucocerebrosidase activity, enhancing cognitive functions in patients with PD [57]. For all these reasons, it would be interesting to analyze the levels of these lipid metabolites to understand if, as well as PC and PE, they are decreased in the liver of these GS-PD mice.

Some limitations of this study should be addressed. To reduce the effect of estrogen modulations on metabolism, this study was performed only in male mice; however, a comparison could be made between the two genders. In addition, some metabolites of interest, such as ceramides and sphingomyelin, could not be analyzed, hence additional work may improve the information we have provided. The number of mice analyzed per group could be higher to enhance the robustness of the results; however, due to the high cost of this type of analysis in two different tissue samples and in three experimental groups, this sample size has not been allowed to increase. Overall, we intend to shed light on the general and specific metabolic changes in non-nervous tissues and thus understand if the liver may be at least partially responsible for the appearance of this neurodegenerative disease; however, it is essential to carry out experiments aimed at demonstrating any causal relationship between the liver disease, alteration, appearance and development of PD.

## Data Availability

The data that support the findings of this study are available from the corresponding author upon reasonable request.

## Supplementary Materials

The following supporting information can be downloaded at: https://www.mdpi.com/article/10.3390/cells12050806/s1, Figure S1: Correlation analysis between the changes observed in striatal neurons of the genetic model of PD and acute intoxication compared to healthy mice (Control). Figure S2: Correlation analysis between the changes observed in hepatic cells of the genetic model of PD and acute intoxication compared to healthy mice (Control). Figure S3: Summary with the models used and the main metabolic results obtained in this work. Table S1: Metabolite changes in striatum cells from control and PD mouse models (GS-PD or ai-PD). Table S2: Metabolite changes in hepatic cells from control and PD mouse models (GS-PD or ai-PD).
