# Supplementary material for: Changes in Liver Lipidomic Profile in G2019S-LRRK2 Mouse Model of Parkinson’s Disease
Source: Cells. 2023 Mar 4;12(5):806. doi: 10.3390/cells12050806 (PMC10000529; doi:10.3390/cells12050806)
Supplement: Supplementary file 1 [file cells-12-00806-s001.zip › cells-2157961-SI.pdf]

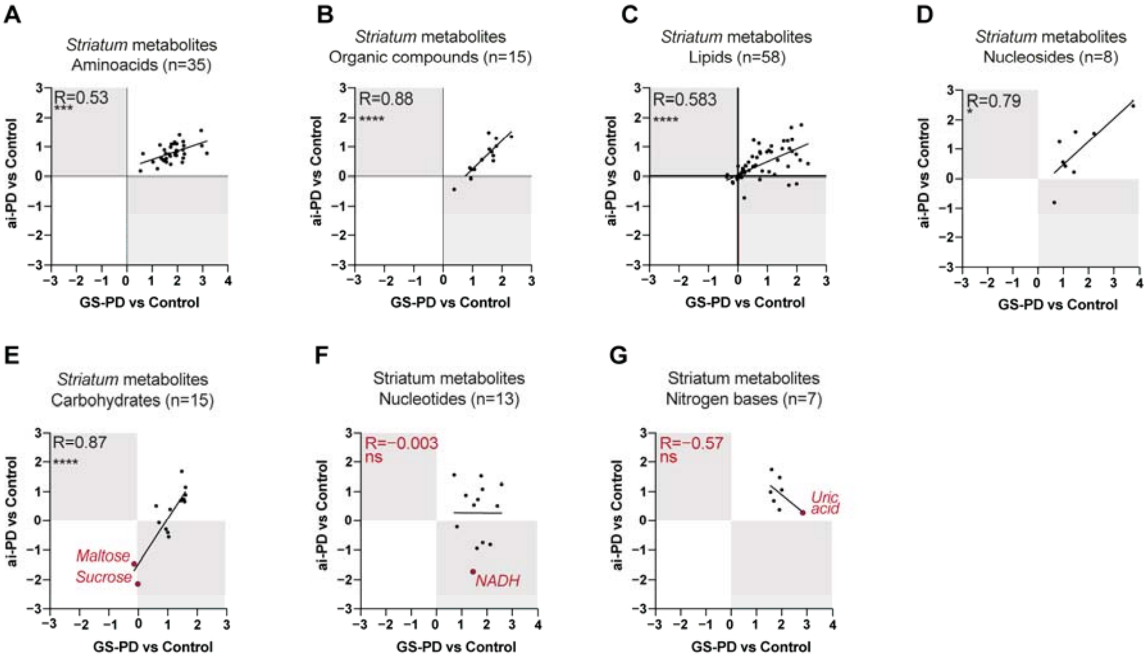

Figure S1: Correlation analysis between the changes observed in striatal neurons of the genetic model of PD (GS-PD) and acute intoxication (ai-PD) compared to healthy mice (Control). Striatal metabolites were analyzed by divisions into metabolic routes: amino acids (A), organic compounds (B), lipids (C), nucleosides (D), carbohydrates (E), nucleotides (F) and nitrogen bases (G). Statistical analysis was performed by obtaining p values (ns: non-significant; *** ($p<0.001$), **** ($p<0.0001$)), and Pearson's correlation coefficients (R) between the observed changes.

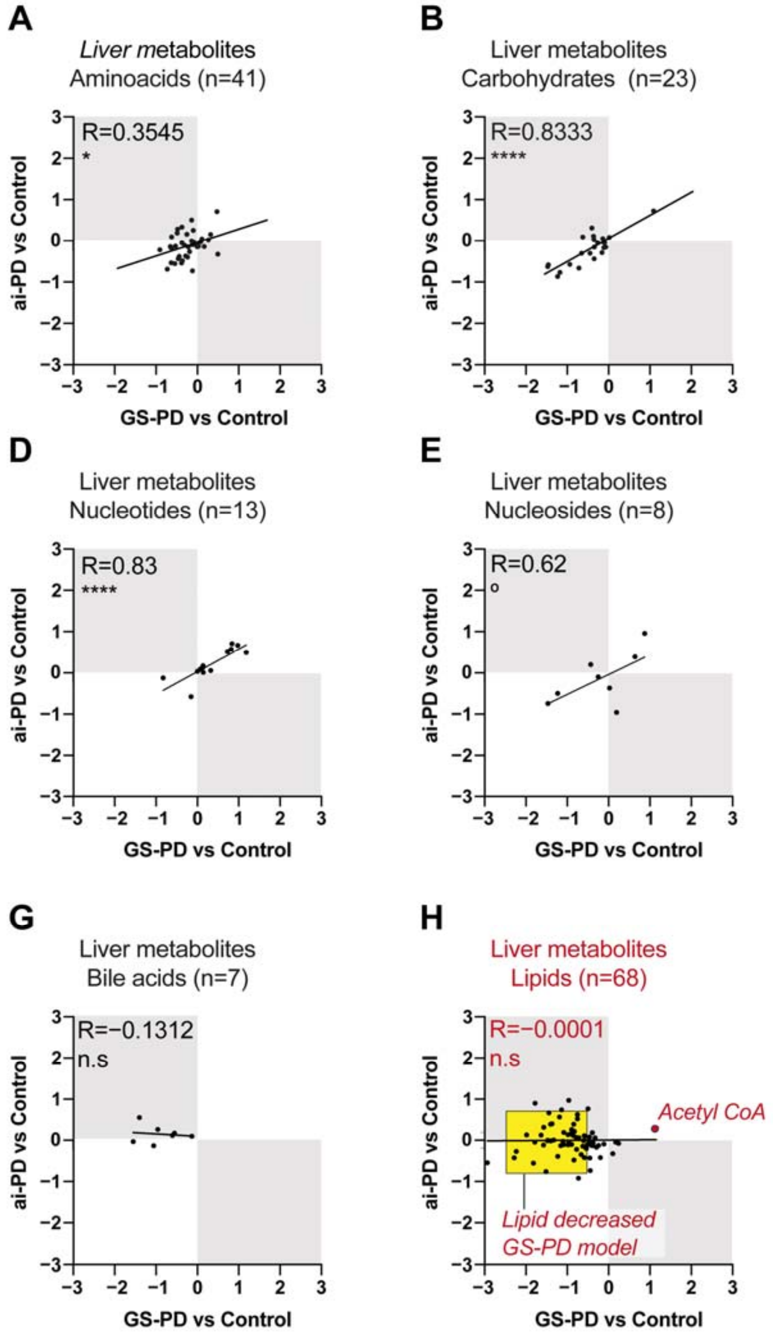

Figure S2: Correlation analysis between the changes observed in hepatic cells of the genetic model of PD (GS-PD) and acute intoxication (ai-PD) compared to healthy mice (Control). Metabolites were analyzed by divisions into metabolic routes: amino acids (A), carbohydrates (B), organic compounds (C), nucleotides (D), nucleosides (E), nitrogenous bases (F), bile acids (G) and lipids (H). Statistical analysis was performed by obtaining p values (n.s: non-significant; º ($p<0.1$),* ($p<0.05$), ** ($p<0.01$), **** ($p<0.0001$)), and Pearson's correlation coefficients (R) between the observed changes.

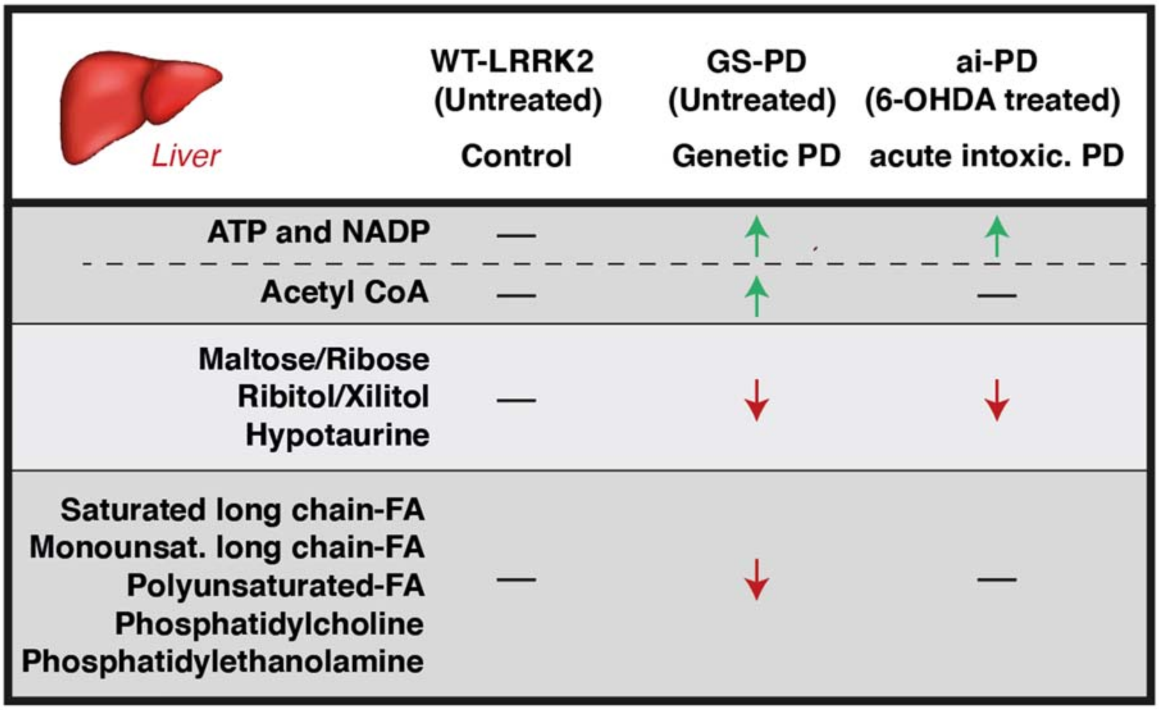

Figure S3: Summary with the models used and the main metabolic results obtained in this work. Green arrows indicate an increase and red arrows indicate a decrease in levels compared to the untreated control mice. 6-OHDA, 6-hydroxydopamine; ai, acute intoxication; ATP, adenosine triphosphate; FA, fatty acids; GS, *LRRK2* G2019S mutation; NADH, nicotinic amide adenine dinucleotide; NADP, Nicotinamide adenine dinucleotide phosphate; PD, Parkinson disease.

Class	Name	Control 1	Control 2	Control 3	Control 4	Control 5	N	AVER	SEM	GS-PD 1	GS-PD 2	GS-PD 3	GS-PD 4	GS-PD 5	N	AVERAGE	SEM	ai-PD 1	ai-PD 2	ai-PD 3	ai-PD 4	ai-PD 5	N	AVERAGE	SEM	log2FC	TTEST	log2FC	TTEST
Amino acids	Dimethylarginine	-0,47	-6,65	-4,38	-0,03	-0,93	5,00	-2,49	1,29	-1,28	-1,61	0,93	0,73	-0,81	5,00	-0,41	0,52	-0,71	-1,00	-0,59	-2,52	-0,58	5,00	-1,08	0,37	2,08	0,17	1,41	0,32
	Pantothenic acid	-1,66	-5,28	-2,82	0,35	-0,79	5,00	-2,04	0,96	-0,75	-0,73	0,78	0,34	-0,45	5,00	-0,16	0,31	-2,25	-0,87	0,51	-1,40	-0,73	5,00	-0,95	0,45	1,88	0,10	1,09	0,34
	2-aminoadipic acid	-1,03	-4,86	-3,14	0,75	-0,08	5,00	-1,67	1,03	0,00	0,29	1,25	1,08	0,17	5,00	0,56	0,25	-1,58	-0,33	0,58	-1,88	0,67	5,00	-0,51	0,53	2,23	0,07	1,16	0,34
	Trimethyl-lysine	-0,32	-4,88	-3,69	0,36	-0,41	5,00	-1,79	1,04	-1,27	-1,24	0,12	0,88	-0,89	5,00	-0,48	0,42	-0,72	-0,82	0,01	-2,00	-0,17	5,00	-0,74	0,35	1,31	0,28	1,05	0,37
	Isoleucine	-0,97	-5,38	-2,64	1,01	-0,23	5,00	-1,64	1,11	-0,26	-0,25	0,96	1,03	0,16	5,00	0,33	0,28	-1,59	-0,22	0,64	-1,47	-0,12	5,00	-0,55	0,43	1,97	0,12	1,09	0,38
	Alanine	-0,77	-5,70	-2,32	1,22	-0,44	5,00	-1,60	1,17	-0,22	0,20	0,36	0,37	0,11	5,00	0,17	0,11	-1,17	-0,46	0,36	-1,13	-0,23	5,00	-0,53	0,29	1,77	0,17	1,07	0,40
	Leucine	-0,79	-4,57	-2,06	1,04	-0,14	5,00	-1,30	0,96	-0,43	-0,30	0,88	0,85	-0,10	5,00	0,18	0,28	-0,98	-0,10	0,54	-1,40	-0,17	5,00	-0,42	0,34	1,48	0,18	0,88	0,41
	Proline	-1,29	-8,36	-4,20	1,26	-0,66	5,00	-2,65	1,67	-0,41	-0,59	1,58	0,91	-0,04	5,00	0,29	0,41	-3,12	-0,36	0,60	-2,53	-0,06	5,00	-1,09	0,73	2,94	0,13	1,56	0,42
	Arginine	0,46	-3,66	-2,26	-0,17	-0,29	5,00	-1,18	0,77	-0,98	-1,71	-0,36	0,70	-0,38	5,00	-0,54	0,40	-0,06	0,12	0,40	-2,60	-0,02	5,00	-0,43	0,55	0,64	0,48	0,75	0,45
	Serine	-0,82	-6,72	-2,80	1,18	-0,03	5,00	-1,84	1,38	-0,80	-0,17	0,36	0,60	0,29	5,00	0,05	0,25	-2,25	-0,17	0,74	-1,61	-0,20	5,00	-0,70	0,54	1,89	0,21	1,14	0,46
	5-aminovaleric acid	-1,08	-6,24	-2,21	1,33	-0,43	5,00	-1,73	1,27	-0,40	0,01	0,93	0,79	-0,14	5,00	0,24	0,26	-1,43	-1,31	0,19	-1,44	-0,31	5,00	-0,86	0,34	1,96	0,17	0,86	0,53
	Glutamic acid	-1,15	-5,94	-2,52	1,42	-0,41	5,00	-1,72	1,23	-0,43	-0,01	1,12	0,81	-0,10	5,00	0,28	0,29	-1,38	-1,36	0,12	-1,43	-0,37	5,00	-0,88	0,32	2,00	0,15	0,84	0,53
	N-acetylglutamine	-0,74	-7,53	-3,75	0,93	-0,97	5,00	-2,41	1,48	-1,97	-1,50	2,32	0,95	-0,60	5,00	-0,16	0,79	-1,75	-1,32	0,12	-3,14	-0,85	5,00	-1,39	0,54	2,25	0,22	1,02	0,53
	Citrulline	-0,65	-7,61	-2,52	0,33	-0,24	5,00	-2,14	1,45	-0,73	-0,87	-0,25	0,18	-0,35	5,00	-0,40	0,19	-2,69	-0,45	0,45	-2,27	-0,85	5,00	-1,16	0,58	1,73	0,27	0,98	0,55
	Valine	-1,15	-4,66	-2,37	0,73	-0,47	5,00	-1,58	0,92	-0,73	-0,62	0,44	0,15	-0,36	5,00	-0,23	0,23	-1,79	-0,79	0,12	-1,76	-0,72	5,00	-0,99	0,36	1,36	0,19	0,60	0,56
	Tyrosine	-1,30	-6,58	-2,76	1,58	-0,55	5,00	-1,92	1,36	-0,30	-0,39	1,54	1,12	-0,49	5,00	0,29	0,43	-2,77	-1,10	0,90	-2,07	-0,16	5,00	-1,04	0,66	2,22	0,16	0,88	0,58
	Creatine	-0,45	-5,09	-1,98	1,43	-0,13	5,00	-1,24	1,10	0,23	0,32	0,96	1,12	0,72	5,00	0,67	0,17	-1,91	-0,47	0,95	-1,34	0,08	5,00	-0,54	0,51	1,91	0,13	0,70	0,58
	Dimethylglycine	-0,06	-4,63	-2,61	-0,21	-0,69	5,00	-1,64	0,87	-0,60	-1,57	0,66	1,18	-0,07	5,00	-0,08	0,48	-1,33	-0,96	0,51	-2,75	-0,74	5,00	-1,05	0,53	1,56	0,16	0,59	0,58
	4-hydroxyproline	-1,03	-6,51	-2,77	1,20	-0,79	5,00	-1,98	1,29	-0,78	-1,07	-0,02	0,01	-0,37	5,00	-0,44	0,21	-1,92	-1,21	-0,47	-1,63	-0,89	5,00	-1,22	0,26	1,54	0,28	0,76	0,58
	Aspartic acid	-0,84	-6,16	-2,31	1,06	-0,36	5,00	-1,72	1,23	-0,52	-0,71	0,44	0,71	-0,15	5,00	-0,04	0,27	-2,34	-1,04	0,41	-1,70	-0,26	5,00	-0,99	0,49	1,68	0,22	0,74	0,59
	Glutamine	-1,62	-8,38	-3,01	1,07	-0,14	5,00	-2,42	1,64	-0,11	0,16	1,62	1,39	-0,30	5,00	0,55	0,40	-4,01	-1,42	0,92	-2,28	-0,19	5,00	-1,40	0,85	2,97	0,12	1,02	0,60
	5-oxoproline	-0,82	-4,39	-2,18	0,99	-0,56	5,00	-1,39	0,90	-0,58	-0,24	1,07	0,42	-0,31	5,00	0,07	0,30	-1,89	-0,85	0,16	-1,45	-0,36	5,00	-0,88	0,37	1,47	0,16	0,52	0,61
	Ornithine	-0,87	-5,95	-2,12	1,29	0,14	5,00	-1,50	1,25	0,02	0,04	0,61	1,31	0,13	5,00	0,42	0,25	-2,99	-0,35	1,30	-2,07	0,35	5,00	-0,75	0,79	1,92	0,17	0,75	0,62
	Histidine	-0,85	-6,64	-2,47	1,21	-0,65	5,00	-1,88	1,33	-0,22	-0,13	0,75	1,41	0,16	5,00	0,40	0,30	-2,86	-0,87	1,03	-2,41	-0,59	5,00	-1,14	0,69	2,28	0,13	0,74	0,63
	Homoserine	-0,74	-6,09	-2,14	1,04	-0,20	5,00	-1,62	1,23	-0,62	-0,18	0,34	0,55	0,37	5,00	0,09	0,22	-2,68	-0,27	0,56	-1,94	-0,42	5,00	-0,95	0,59	1,71	0,21	0,67	0,63
	Threonine	-0,89	-4,84	-2,37	1,04	-0,35	5,00	-1,48	1,00	-0,60	-0,34	0,11	0,69	-0,16	5,00	-0,06	0,22	-2,55	-0,78	0,30	-1,61	0,00	5,00	-0,93	0,52	1,42	0,20	0,55	0,64
	N6-acetyllysine;N2-acetyllysine	-0,59	-6,09	-4,35	0,75	-0,63	5,00	-2,18	1,29	-1,17	-1,65	0,04	1,60	0,07	5,00	-0,22	0,57	-2,15	-1,48	0,06	-3,43	-0,44	5,00	-1,49	0,62	1,96	0,20	0,69	0,64
	Glycine	-0,77	-4,27	-2,37	0,82	-0,43	5,00	-1,40	0,88	-0,20	-0,65	1,45	0,60	-0,40	5,00	0,16	0,38	-1,77	-0,40	0,20	-1,98	-0,75	5,00	-0,94	0,41	1,56	0,14	0,47	0,64
	3-methylhistidine	0,01	-4,45	-2,76	0,35	0,06	5,00	-1,36	0,96	-0,92	-1,34	0,21	0,88	-0,47	5,00	-0,33	0,40	-0,52	-0,83	0,09	-2,53	-0,64	5,00	-0,88	0,44	1,03	0,35	0,47	0,66
	Lysine	-0,79	-6,27	-2,54	1,27	-0,53	5,00	-1,77	1,28	-0,14	-0,08	0,83	1,53	0,18	5,00	0,46	0,32	-3,03	-0,87	1,08	-2,43	-0,59	5,00	-1,17	0,73	2,23	0,13	0,60	0,69
	Tryptophan	-1,73	-7,76	-4,69	2,51	-0,50	5,00	-2,44	1,76	-0,37	0,19	1,89	2,29	-0,34	5,00	0,73	0,57	-4,63	-1,72	1,38	-3,29	-0,12	5,00	-1,68	1,08	3,17	0,13	0,76	0,72
	Asparagine	-1,35	-3,62	-2,64	1,88	-0,58	5,00	-1,26	0,94	0,20	0,14	0,56	1,47	-0,02	5,00	0,47	0,27	-3,02	-1,08	0,73	-1,30	0,32	5,00	-0,87	0,66	1,73	0,12	0,39	0,74
	N-acetylaspartic acid	-1,41	-5,44	-2,57	2,77	-0,80	5,00	-1,49	1,33	0,16	0,18	1,19	2,07	-0,08	5,00	0,70	0,41	-2,96	-1,50	0,95	-1,41	-0,25	5,00	-1,03	0,66	2,19	0,15	0,46	0,76
	Betaine;Valine	0,45	-2,78	-1,55	0,38	-0,54	5,00	-0,81	0,61	-0,86	-0,96	-0,20	0,94	-0,26	5,00	-0,27	0,34	-0,88	-0,41	-0,06	-1,45	-0,41	5,00	-0,64	0,24	0,54	0,46	0,17	0,81
	Creatinine	0,07	-4,22	-2,78	0,91	-0,27	5,00	-1,26	0,96	-1,02	-1,14	0,70	1,28	-0,07	5,00	-0,05	0,47	-1,22	-0,91	0,18	-2,58	-0,55	5,00	-1,02	0,45	1,21	0,29	0,24	0,82
Bases	Guanine	-0,35	-5,12	-4,61	-0,57	-0,61	5,00	-2,25	1,07	-2,49	-1,94	0,41	1,23	-0,46	5,00	-0,65	0,70	-1,99	-0,21	0,89	-2,17	0,95	5,00	-0,50	0,68	1,60	0,25	1,75	0,20
	Uracil	-0,61	-3,81	-2,17	0,38	-0,55	5,00	-1,35	0,74	-0,34	-0,17	0,85	0,60	0,10	5,00	0,21	0,23	-1,03	0,05	0,49	-1,05	-0,21	5,00	-0,35	0,30	1,56	0,08	1,00	0,25
	Xanthine	-0,31	-6,07	-3,20	0,32	-0,45	5,00	-1,94	1,20	-0,82	-0,59	0,49	0,82	-0,01	5,00	-0,02	0,31	-2,09	-0,35	1,18	-1,10	0,03	5,00	-0,47	0,55	1,92	0,16	1,47	0,30
	Adenine	-0,68	-3,69	-2,06	0,48	-0,87	5,00	-1,36	0,71	-0,10	0,00	0,85	1,07	-0,21	5,00	0,32	0,26	-1,52	-0,88	0,33	-0,98	-0,30	5,00	-0,67	0,31	1,69	0,06	0,70	0,40
	Hypoxanthine	-0,43	-6,11	-2,64	0,51	-0,55	5,00	-1,84	1,18	-0,56	-0,28	0,67	0,83	0,13	5,00	0,16	0,27	-2,07	-0,51	1,05	-2,04	-0,30	5,00	-0,77	0,59	2,00	0,14	1,07	0,44
	Cytosine	1,04	-1,64	-0,31	0,89	-0,09	5,00	-0,02	0,48	0,37	4,35	0,30	4,64	-0,22	5,00	1,89	1,07	0,23	-0,42	1,01	0,42	0,63	5,00	0,37	0,24	1,91	0,14	0,39	0,49
	Uric acid	-1,60	-5,02	-5,57	1,22	-0,93	5,00	-2,38	1,28	0,13	-0,18	1,03	1,45	-0,13	5,00	0,46	0,33	-3,70	-1,34	0,76	-1,95	-4,21	5,00	-2,09	0,89	2,84	0,06	0,29	0,86
Carbohydrates	Sucrose	1,82	-0,05	2,29	-1,25	-1,68	5,00	0,23	0,80	5,95	0,69	-1,76	-2,09	-1,75	5,00	0,21	1,52	-1,68	-1,63	-1,69	-2,46	-2,11	5,00	-1,92	0,16	-0,02	0,99	-2,14	0,03
	Maltose;Trehalose	-0,81	-1,80	-1,85	0,68	-1,31	5,00	-1,02	0,46	-1,60	-1,43	-0,60		-1,01	4,00	-1,16	0,22	-3,07	-1,49		-3,34	-2,00	4,00	-2,48	0,44	-0,14	0,81	-1,46	0,06
	Lactose	0,34	-4,90	-3,35	0,35	-0,46	5,00	-1,60	1,07	-0,03	0,30	-0,25	-0,53	-0,11	5,00	-0,13	0,13	0,61	0,60	-0,45	-0,55	0,15	5,00	0,07	0,25	1,48	0,21	1,68	0,17
	Ribose;Ribulose	-0,33	-3,50	-2,00	0,29	-0,35	5,00	-1,18	0,69	-0,05	0,11	0,53	1,07	0,47	5,00	0,43	0,19	-0,98	0,08	1,01	-0,57	0,34	5,00	-0,02	0,35	1,60	0,06	1,15	0,18
	Arabinose	-0,85	-4,49	-2,44	0,66	-0,46	5,00	-1,52	0,89	-0,24	-0,34	0,51	0,57	-0,07	5,00	0,09	0,19	-1,79	-0,75	0,33	-0,68	-0,22	5,00	-0,62	0,35	1,60	0,12	0,89	0,38
	Glycerol	-0,70	-2,73	-1,05	0,55	-0,61	5,00	-0,91	0,53	-0,73	-0,48	0,18	0,12	-0,58	5,00	-0,30	0,19	-0,60	0,09	0,07	-1,02	-0,49	5,00	-0,39	0,21	0,61	0,31	0,52	0,39
	Arabitol	-1,22	-4,33	-2,89	0,34	-0,73	5,00	-1,76	0,82	-0,33	-0,81	0,25	0,35	-0,55	5,00	-0,22	0,23	-2,04	-1,14	0,14	-1,56	-0,53	5,00	-1,02	0,38	1,55	0,11	0,74	0,44
	Xylitol	-0,69	-4,13	-2,20	1,10	-0,67	5,00	-1,32	0,88	-0,09	-0,38	0,36	0,90	0,05	5,00	0,17	0,22	-1,78	-0,48	0,55	-1,09	-0,12	5,00	-0,58	0,40	1,48	0,14	0,73	0,47
	Ribitol	-0,92	-4,00	-1,66	0,69	0,05	5,00	-1,17	0,82	-0,02	0,21	-0,15	1,02	0,34	5,00	0,28	0,20	-1,78	-0,70	0,54	-0,77	0,36	5,00	-0,47	0,42	1,44	0,12	0,70	0,47
	Galactitol;Sorbitol;Mannitol	-2,29	-1,18	-2,86	-2,38	-1,86	5,00	-2,12	0,28	-2,08	-2,12	-0,99	1,81	-2,03	5,00	-1,08	0,75	-2,86	-2,59	-0,05	-4,89	-2,94	5,00	-2,67	0,77	1,03	0,24	-0,55	0,52
	Erythritol	-0,92	-4,77	-2,36	1,06	-0,72	5,00	-1,54	0,97	-0,38	-0,43	0,61	0,49	-0,13	5,00	0,03	0,22	-1,88	-1,07	0,28	-1,20	-0,51	5,00	-0,88	0,36	1,57	0,15	0,66	0,54
	Threonic acid	-0,66	-3,22	-1,95	1,05	-0,45	5,00	-1,05	0,72	-0,51	-0,60	0,44	0,56	0,30	5,00	0,04	0,25	-1,77	-0,83	0,71	-1,04	-0,31	5,00	-0,65	0,41	1,08	0,19	0,40	0,64
	Galactose;Glucose;Mannose	-2,01	-0,99	-3,18	-2,17	-1,69	5,00	-2,01	0,36	-2,18	-2,27	-0,62	1,91	-1,85	5,00	-1,00	0,79	-2,49	-2,21	0,52	-4,94	-2,93	5,00	-2,41	0,87	1,00	0,28	-0,40	0,68
	Fructose	-1,23	-3,30	-1,30	0,94	-0,22	5,00	-1,02	0,70	-0,36	-0,42	0,12	0,77	-0,50	5,00	-0,08	0,24	-2,71	-1,23	0,37	-1,88	-1,09	5,00	-1,31	0,51	0,94	0,24	-0,29	0,75
	Xylose	0,55	-4,03	-1,82	1,37	-0,82	5,00	-0,95	0,95	-0,21	-0,66	0,51	-0,64	-0,22	5,00	-0,24	0,21	-2,27	-0,91	-0,07	-1,40	-0,41	5,00	-1,01	0,39	0,70	0,49	-0,06	0,95
Fatty Acyls	Heptadecenoic acid	0,06	-0,22	-0,20	0,25	-0,71	5,00	-0,17	0,16	-0,55	-0,06	0,65	0,91	0,02	5,00	0,19	0,26	0,23	0,01	0,49	0,37	0,45	5,00	0,31	0,09	0,36	0,28	0,47	0,03
	Stearic acid	-0,45	-0,42	-0,58	0,05	-0,36	5,00	-0,35	0,11	-0,19	-0,31	0,05	0,35	-0,30	5,00	-0,08	0,13	-0,08	-0,23	0,28	-0,17	0,17	5,00	-0,01	0,10	0,27	0,14	0,35	0,04
	Arachidic acid	-0,22	-0,34	-0,41	-0,03	-0,27	5,00	-0,25	0,06	-0,21	-0,22	-0,01	0,25	-0,26	5,00	-0,09	0,10	-0,05	-0,20	0,29	-0,17	0,15	5,00	0,00	0,09	0,16	0,19	0,26	0,05
	Palmitic acid	-0,26	-0,58	-2,65	0,08	-1,73	5,00	-1,03	0,51	-1,37	-0,48	0,15	0,52	-0,34	5,00	-0,31	0,32	-0,12	-0,15	0,57	-0,16	0,32	5,00	0,09	0,15	0,72	0,26	1,12	0,07
	Docosenoic acid	-0,33	-1,09	-1,03	-0,12	-0,99	5,00	-0,71	0,20	-1,00	-0,53	0,11	0,64	-0,32	5,00	-0,22	0,28	-0,55	-0,25	0,80	-0,42	0,16	5,00	-0,05	0,24	0,49	0,19	0,66	0,07
	Pentadecanoic acid	0,25	-0,27	-0,02	0,10	-0,60	5,00	-0,11	0,15	-1,44	0,18	0,21	0,50	0,04	5,00	-0,10	0,34	0,27	-0,16	0,31	0,35	0,37	5,00	0,23	0,10	0,01	0,99	0,34	0,10
	Nonadecanoic acid	-0,15	-0,67	-0,63	-0,02	0,07	5,00	-0,28	0,15	-0,04	-0,19	-0,06	0,27	-0,10	5,00	-0,02	0,08	-0,09	0,00	0,53	-0,18	0,12	5,00	0,08	0,12	0,26	0,18	0,36	0,11
	Undecanoic acid	-0,20	0,41	-3,59	-1,46	-1,10	5,00	-1,19	0,68	-1,44	-2,46	0,41	0,03	0,21	5,00	-0,65	0,56	0,15	-0,58	0,35	0,14	0,15	5,00	0,04	0,16	0,54	0,56	1,23	0,12
	Docosahexaenoic acid	-1,01	-5,38	-2,62	0,38	-1,26	5,00	-1,98	0,97	-0,86	-1,22	0,89	1,57	-1,17	5,00	-0,16	0,58	-0,85	-1,35	1,13	-0,90	0,42	5,00	-0,31	0,47	1,82	0,15	1,67	0,16
	Behenic acid	0,18	-0,31	-0,34	0,00	-0,46	5,00	-0,19	0,12	-0,19	-0,26	0,01	0,36	0,06	5,00	0,00	0,11	0,10	0,09	0,27	-0,37	0,18	5,00	0,06	0,11	0,18	0,29	0,24	0,17
	Eicosadienoic acid	-0,62	-1,95	-1,59	0,03	-1,92	5,00	-1,21	0,39	-0,94	-0,90	0,46	0,61	-1,48	5,00	-0,45	0,41	-1,01	-0,70	0,73	-1,13	-0,07	5,00	-0,44	0,34	0,76	0,22	0,77	0,18
	AcetylCoA	-0,32	-4,49	-2,06	-0,11	-1,49	5,00	-1,70	0,79	0,15	0,34	0,34	-0,06	-0,79	5,00	0,00	0,21	-0,63	-0,52	-0,48	-1,08	0,24	5,00	-0,49	0,21	1,69	0,07	1,20	0,18
	Hydroxydodecanoic acid*	0,10	0,00	-0,03	-0,03	-0,08	5,00	-0,01	0,03	-0,23	-0,06	-0,08	0,07	0,09	5,00	-0,04	0,06	-0,12	-0,01	-0,01	-0,10	-0,07	5,00	-0,06	0,02	-0,03	0,63	-0,05	0,18
	Eicosenoic acid	-0,40	-1,38	-1,13	0,13	-0,83	5,00	-0,72	0,27	-0,74	-0,78	0,45	1,18	-0,48	5,00	-0,08	0,39	-0,75	-0,41	1,16	-0,74	0,30	5,00	-0,09	0,37	0,65	0,21	0,63	0,20
	Eicosatrienoic acid	-0,66	-2,54	-1,67	0,13	-0,68	5,00	-1,08	0,46	-0,97	-0,70	0,62	1,26	-0,69	5,00	-0,10	0,44	-0,80	-0,93	1,08	-0,92	0,25	5,00	-0,26	0,40	0,99	0,16	0,82	0,22
	Linoleic acid	0,10	-0,20	-0,14	-0,09	0,01	5,00	-0,06	0,05	0,06	-0,15	0,10	0,22	-0,02	5,00	0,04	0,06	0,06	-0,08	0,19	-0,01	0,00	5,00	0,03	0,05	0,10	0,24	0,09	0,22
	Dodecenoic acid	-0,13	0,20	-3,52	-0,20	-0,01	5,00	-0,73	0,70	-0,63	-0,21	0,85	-0,20	0,26	5,00	0,01	0,25	0,06	-0,51	0,75	0,09	0,08	5,00	0,09	0,20	0,75	0,34	0,83	0,29
	Arachidonic acid	-1,13	-5,08	-2,93	0,85	-1,19	5,00	-1,90	1,00	-0,97	-0,59	1,02	1,57	-1,30	5,00	-0,05	0,57	-1,93	-1,48	1,08	-1,10	0,16	5,00	-0,65	0,55	1,84	0,15	1,24	0,31
	Mandelic acid*	0,47	0,28	NA	0,52	0,10	4,00	0,35	0,10	NA	-0,06	0,24	0,39	0,15	4,00	0,18	0,09	0,33	-0,18	0,15	-0,24	0,59	5,00	0,13	0,16	-0,17	0,26	-0,21	0,31
	Pentadecenoic acid	-0,25	0,12	-0,48	0,02	-0,10	5,00	-0,14	0,10	0,07	0,09	-0,03	0,28	0,15	5,00	0,11	0,05	-0,08	-0,29	0,19	0,21	0,05	5,00	0,01	0,09	0,25	0,07	0,15	0,32
	Docosatetraenoic acid	-1,13	NA	-2,93	0,12	-1,63	4,00	-1,39	0,63	-1,56	-0,92	0,57	1,37	-1,16	5,00	-0,34	0,56	-1,85	-1,10	1,47	-1,63	0,51	5,00	-0,52	0,65	1,05	0,25	0,87	0,37
	Oleic acid;Elaidic acid	-0,36	-2,00	-1,06	-0,06	-0,62	5,00	-0,82	0,34	-0,59	-0,53	-0,10	0,28	-0,59	5,00	-0,31	0,17	-0,82	-0,50	0,36	-0,98	-0,56	5,00	-0,50	0,23	0,51	0,21	0,32	0,46
	Palmitoleic acid	-0,42	-2,09	-1,48	0,18	-0,56	5,00	-0,88	0,40	-0,70	-0,59	0,37	-0,09	-0,37	5,00	-0,27	0,19	-0,98	-0,08	0,25	-1,21	-0,50	5,00	-0,51	0,27	0,60	0,22	0,37	0,47
	Carnitine C14:0	-1,93	-3,44	-3,08	1,46	-1,41	5,00	-1,68	0,87	-2,55	-0,55	0,40	2,13	-0,27	5,00	-0,17	0,76	-3,64	-0,36	1,41	-1,83	0,67	5,00	-0,75	0,90	1,51	0,23	0,93	0,48
	Docosanedioic acid	0,19	-0,09	-0,07	0,02	-0,17	5,00	-0,03	0,06	0,07	0,01	0,06	0,12	0,02	5,00	0,06	0,02	0,09	-0,02	0,05	-0,05	0,05	5,00	0,02	0,03	0,08	0,25	0,05	0,49
	Hexadecadienoic acid	-0,02	-0,08	-0,54	0,13	-0,94	5,00	-0,29	0,20	-0,40	0,15	0,01	0,24	0,44	5,00	0,09	0,14	0,31	-0,69	0,14	0,03	-0,28	5,00	-0,10	0,18	0,38	0,16	0,19	0,49
	Undecanedioic acid	0,00	0,11	-0,17	0,04	-0,14	5,00	-0,03	0,05	0,00	-0,90	-0,02	-1,21	0,18	5,00	-0,39	0,28	-0,09	-0,03	0,12	0,13	-0,05	5,00	0,02	0,04	-0,36	0,24	0,05	0,51
	Glutarylcarnitine	-0,23	-7,40	-3,66	1,22	-0,69	5,00	-2,15	1,53	-2,30	-2,00	-1,84	1,22	-0,16	5,00	-1,02	0,67	-1,98	-1,11	0,50	-2,86	-0,20	5,00	-1,13	0,60	1,14	0,52	1,02	0,55
	Malonylcarnitine	-0,28	-6,15	-4,42	0,62	-0,83	5,00	-2,21	1,30	-1,65	-1,19	0,28	1,08	-0,37	5,00	-0,37	0,49	-2,38	-0,92	0,29	-3,11	-0,73	5,00	-1,37	0,61	1,84	0,22	0,84	0,57
	Myristic acid	-0,22	-0,01	-0,12	0,04	-0,06	5,00	-0,07	0,04	0,02	-0,05	-0,09	0,29	-0,09	5,00	0,02	0,07	-0,25	-0,21	0,13	0,17	0,07	5,00	-0,02	0,09	0,09	0,31	0,06	0,58
	Dodecenedioic acid	-0,15	0,09	0,16	0,11	-0,72	5,00	-0,10	0,16	-0,76	0,28	0,24	0,49	0,36	5,00	0,12	0,22	0,20	-0,05	-0,41	-0,03	0,33	5,00	0,01	0,13	0,22	0,45	0,11	0,62
	Carnitine C4:0(OH)	-0,57	-6,81	-3,63	0,67	-0,43	5,00	-2,16	1,37	-1,22	-1,90	0,86	1,43	-0,03	5,00	-0,17	0,62	-2,55	-1,12	0,25	-3,12	-0,53	5,00	-1,41	0,63	1,98	0,22	0,74	0,63
	Docosapentaenoic acid	-1,59	NA	NA	0,65	-1,46	3,00	-0,80	0,72	-3,09	-1,62	1,04	2,27	-1,51	5,00	-0,58	0,98	-2,04	-3,72	1,94	-3,52	-0,25	5,00	-1,52	1,06	0,22	0,88	-0,72	0,65
	Carnitine	-0,20	-5,03	-2,98	0,74	-0,57	5,00	-1,61	1,05	-1,40	-1,42	0,65	1,14	-0,22	5,00	-0,25	0,52	-1,70	-0,83	0,34	-2,74	-0,39	5,00	-1,07	0,53	1,35	0,28	0,54	0,66
	Carnitine C6:0	-2,31	NA	-5,40	0,32	-0,88	4,00	-2,07	1,23	-0,68	-2,22	1,72	1,63	-0,18	5,00	0,05	0,74	NA	-2,52	-0,91	NA	-1,14	3,00	-1,52	0,50	2,12	0,17	0,54	0,74
	Tetradecadienoic acid	-0,12	0,64	-0,56	0,35	-0,34	5,00	-0,01	0,22	-0,50	-0,07	-0,23	0,67	0,03	5,00	-0,02	0,19	-0,10	-0,04	0,46	-0,19	-0,66	5,00	-0,10	0,18	-0,01	0,97	-0,10	0,74
	Sebacic acid	-0,09	-0,02	-2,06	-0,14	-0,02	5,00	-0,47	0,40	-0,88	-2,36	0,09	-0,42	0,35	5,00	-0,64	0,48	-1,34	-0,13	0,53	-1,63	-0,64	5,00	-0,64	0,39	-0,18	0,78	-0,18	0,76
	Nonadecenoic acid	0,40	-0,77	-0,97	0,37	-0,24	5,00	-0,24	0,28	-0,39	-0,73	0,44	0,94	-0,68	5,00	-0,08	0,33	-0,70	0,15	1,01	-1,71	0,87	5,00	-0,08	0,51	0,16	0,73	0,17	0,78
	Carnitine C3:0	-1,01	-6,01	-3,83	1,19	-1,42	5,00	-2,22	1,24	-1,34	-1,18	1,09	1,70	0,55	5,00	0,16	0,61	-2,51	-1,42	0,16	-4,50	-0,71	5,00	-1,79	0,81	2,38	0,12	0,42	0,78
	Acetylcarnitine	-0,76	-5,16	-3,37	0,67	-0,53	5,00	-1,83	1,06	-1,48	-1,48	0,97	1,46	-0,09	5,00	-0,12	0,61	-2,18	-1,38	-0,43	-3,05	-0,48	5,00	-1,50	0,50	1,71	0,20	0,33	0,79
	Tetradecanedioic acid	-0,20	0,07	0,10	0,01	-0,11	5,00	-0,03	0,06	-0,58	-0,13	0,15	0,16	0,29	5,00	-0,02	0,15	0,07	-0,07	-0,17	-0,17	0,11	5,00	-0,05	0,06	0,00	0,99	-0,02	0,79
	Carnitine C16:0	-1,29	NA	-2,94	0,95	-0,72	4,00	-1,00	0,80	-2,14	-1,64	0,26	1,99	-0,60	5,00	-0,43	0,73	-3,25	-0,51	1,69	-2,02	0,72	5,00	-0,67	0,90	0,57	0,62	0,33	0,80
	Eicosapentaenoic acid	-0,17	0,16	0,09	0,06	0,08	5,00	0,04	0,06	0,26	0,10	-0,04	0,44	0,07	5,00	0,17	0,08	-0,17	-0,16	0,25	0,15	0,04	5,00	0,02	0,08	0,12	0,26	-0,02	0,83
	Caproic acid	-0,07	0,02	0,07	0,08	0,16	5,00	0,05	0,04	0,08	-0,12	0,38	0,09	-0,01	5,00	0,09	0,08	-0,05	0,06	0,03	0,17	0,01	5,00	0,04	0,04	0,03	0,75	-0,01	0,83
	Carnitine C4:0	-0,93	-6,11	-3,46	1,07	-0,64	5,00	-2,01	1,25	-1,69	-1,38	1,32	1,64	0,04	5,00	-0,02	0,68	-4,02	-1,52	-0,46	-4,80	-0,49	5,00	-2,26	0,91	2,00	0,20	-0,24	0,88
	Carnitine C5:0	-0,60	-6,74	-5,20	1,43	-0,60	5,00	-2,34	1,55	-2,40	-1,53	-0,55	1,47	-0,03	5,00	-0,61	0,66	-4,75	-2,21	0,28	-6,10	-0,36	5,00	-2,63	1,23	1,73	0,33	-0,29	0,89
	Dodecanedioic acid	-0,11	-0,09	0,05	0,04	0,05	5,00	-0,01	0,04	-0,26	-1,36	0,04	-0,52	0,13	5,00	-0,39	0,27	-0,13	0,09	0,08	-0,12	-0,02	5,00	-0,02	0,05	-0,38	0,20	-0,01	0,89
	Myristoleic acid	-0,16	0,23	-0,31	0,02	0,15	5,00	-0,01	0,10	0,08	-0,02	-0,03	0,22	0,13	5,00	0,07	0,05	-0,01	-0,22	0,16	0,13	-0,04	5,00	0,00	0,07	0,09	0,45	0,02	0,90
	Hexadecanedioic acid	-0,01	0,02	0,18	0,27	0,03	5,00	0,10	0,05	-0,38	0,16	0,22	0,28	0,19	5,00	0,10	0,12	-0,04	0,22	0,20	0,23	-0,18	5,00	0,08	0,08	0,00	1,00	-0,01	0,90
	Carnitine C18:1	-1,17	NA	-3,60	0,92	-0,94	4,00	-1,20	0,93	-2,16	-1,45	0,38	2,01	-0,63	5,00	-0,37	0,73	-4,35	-0,71	1,74	-2,28	0,49	5,00	-1,02	1,06	0,83	0,50	0,17	0,91
	Linolenic acid	-0,28	0,28	-0,50	0,32	0,16	5,00	0,00	0,16	-0,10	-0,31	0,49	0,73	-0,17	5,00	0,13	0,20	-0,40	-0,28	0,30	-0,12	0,38	5,00	-0,03	0,16	0,13	0,64	-0,02	0,92
	Carnitine C12:0	-0,26	NA	-4,93	1,45	-0,37	4,00	-1,03	1,37	-1,98	-1,06	-0,26	2,08	-0,19	5,00	-0,28	0,67	-3,98	-0,81	1,12	-2,50	0,56	5,00	-1,12	0,95	0,75	0,62	-0,10	0,95
Lipids	PCae(22:6)	-2,78	NA	-4,02	-0,79	-1,97	4,00	-2,39	0,68	-1,77	-1,39	1,08	1,80	-0,89	5,00	-0,23	0,71	-2,06	-1,10	0,80	-1,70	0,89	5,00	-0,63	0,62	2,16	0,07	1,76	0,10
	PCae(18:1)	-1,41	-4,45	-2,36	0,80	-1,12	5,00	-1,71	0,86	-1,73	-1,14	0,78	1,82	-0,48	5,00	-0,15	0,64	-2,42	-0,69	1,29	-1,42	0,68	5,00	-0,51	0,68	1,56	0,18	1,20	0,31
	PCae(16:0)	-1,29	-3,30	-1,72	1,03	-0,97	5,00	-1,25	0,70	-1,59	-0,74	0,62	1,62	-0,50	5,00	-0,12	0,56	-1,90	-0,72	1,24	-1,18	0,42	5,00	-0,43	0,56	1,13	0,24	0,82	0,38
	PCae(16:1)	-2,93	NA	-6,02	1,10	-1,05	4,00	-2,23	1,51	-3,03	-1,85	1,10	2,04	-0,63	5,00	-0,47	0,93	-3,92	-1,14	1,17	-3,16	0,60	5,00	-1,29	1,00	1,75	0,33	0,94	0,61
	Glycerol-3-phosphate	-1,18	-4,48	-2,90	0,21	-0,24	5,00	-1,72	0,87	-1,13	-1,43	-0,30	-0,25	-0,18	5,00	-0,66	0,26	-2,80	-1,47	-0,34	-1,67	-1,58	5,00	-1,57	0,39	1,06	0,28	0,15	0,88
	Glycerophosphorylcholine	-0,30	-4,73	-2,48	0,45	-0,71	5,00	-1,55	0,93	-0,90	-1,08	-0,12	0,81	-0,22	5,00	-0,30	0,33	-2,02	-1,19	-0,30	-2,95	-1,04	5,00	-1,50	0,45	1,25	0,24	0,05	0,96
Nucleosides	Xanthosine	-0,25	-4,94	-2,15	-0,79	0,02	5,00	-1,62	0,91	-0,31	-1,06	0,31	0,45	0,00	5,00	-0,12	0,27	-1,35	0,06	0,64	-0,02	0,56	5,00	-0,02	0,36	1,50	0,15	1,60	0,14
	Guanosine	0,31	-3,85	-2,18	0,10	-0,69	5,00	-1,26	0,78	-0,32	-0,32	-0,44	-0,44	-0,45	5,00	-0,39	0,03	0,30	0,21	-0,16	-0,44	0,04	5,00	-0,01	0,13	0,87	0,30	1,25	0,15
	Uridine	0,26	-5,07	-5,63	1,42	-7,22	5,00	-3,25	1,72	-0,27	-1,19	1,42	2,34	0,39	5,00	0,54	0,62	-0,96	0,13	0,18	-2,58	-0,58	5,00	-0,76	0,50	3,79	0,07	2,49	0,20
	Succinyladenosine	-1,19	-7,18	-3,93	0,67	-1,03	5,00	-2,53	1,38	-2,17	-1,45	0,48	1,72	-0,08	5,00	-0,30	0,69	-1,35	-1,11	0,33	-2,89	0,08	5,00	-0,99	0,58	2,23	0,19	1,54	0,33
	S-adenosylmethionine	-0,34	NA	-2,63	0,44	-0,84	4,00	-0,84	0,65	-1,33	-1,43	1,08	0,74	0,01	5,00	-0,19	0,52	-1,71	-1,35	-0,08	-4,19	-0,85	5,00	-1,64	0,69	0,66	0,45	-0,79	0,44
	Cytidine	-0,36	-1,86	-3,71	1,36	0,26	5,00	-0,86	0,88	-0,86	0,80	-0,48	1,00	0,24	5,00	0,14	0,36	-0,42	-0,33	0,04	-0,47	-0,35	5,00	-0,30	0,09	1,00	0,32	0,56	0,55
	Adenosine	0,26	-4,11	-1,79	0,28	0,44	5,00	-0,99	0,88	0,01	0,30	0,05	0,37	-0,17	5,00	0,11	0,10	-1,78	-1,14	0,11	0,19	-0,21	5,00	-0,56	0,38	1,10	0,25	0,42	0,67
	Inosine	-0,31	-4,76	-2,54	1,44	-0,34	5,00	-1,30	1,07	-0,35	-0,29	1,00	0,65	-0,33	5,00	0,14	0,29	-2,21	-1,15	0,29	-1,72	-0,63	5,00	-1,09	0,43	1,44	0,23	0,22	0,86
Nucleotides	ATP	-0,74	-2,68	-1,23	-1,13	-0,25	5,00	-1,21	0,41	-0,90	-0,79	-0,71	0,07	-0,20	5,00	-0,51	0,19	-0,73	0,36	0,65	-0,14	1,64	5,00	0,36	0,40	0,70	0,16	1,56	0,03
	NADH	-0,85	-0,68	-0,23	1,61	1,27	5,00	0,22	0,51	1,73	3,21	0,59	2,11	0,75	5,00	1,68	0,48	-2,27	-3,53	0,25	-0,43	-1,51	5,00	-1,50	0,67	1,45	0,07	-1,72	0,07
	NADP	-1,19	-2,32	-1,91	-0,22	-1,06	5,00	-1,34	0,36	-0,42	-0,23	-0,37	0,32	-0,19	5,00	-0,18	0,13	-1,02	-0,50	-0,25	-1,08	0,55	5,00	-0,46	0,30	1,16	0,02	0,88	0,10
	NAD	-2,31	-4,99	-3,57	-0,30	-1,44	5,00	-2,52	0,82	-0,01	-0,34	0,00	0,72	-0,10	5,00	0,05	0,18	-1,97	-1,44	-1,04	-1,70	-0,18	5,00	-1,27	0,31	2,58	0,02	1,26	0,19
	NMN	-0,54	-5,51	-1,05	1,51	-0,80	5,00	-1,28	1,15	0,95	0,36	0,56	0,89	-0,31	5,00	0,49	0,23	0,12	0,09	-0,13	0,43	0,75	5,00	0,25	0,15	1,77	0,17	1,53	0,22
	GDP	-0,42	-4,02	-1,47	0,82	-0,83	5,00	-1,19	0,80	0,08	-0,27	1,82	1,44	0,10	5,00	0,63	0,42	-0,83	0,03	0,44	-0,67	0,54	5,00	-0,10	0,28	1,82	0,08	1,09	0,24
	FAD	-0,68	-3,49	-1,54	0,61	-0,37	5,00	-1,09	0,69	-0,33	0,39	0,90	1,02	-0,03	5,00	0,39	0,26	-1,08	-0,75	0,06	-0,65	-0,30	5,00	-0,54	0,20	1,48	0,08	0,55	0,46
	CMP	-0,62	-4,39	-2,05	0,79	-0,36	5,00	-1,33	0,89	-0,05	0,00	0,58	1,12	-0,09	5,00	0,31	0,24	-1,67	-0,57	0,53	-1,27	0,04	5,00	-0,59	0,40	1,64	0,11	0,74	0,47
	ADP	-1,95	-4,76	-1,32	1,14	-0,26	5,00	-1,43	0,98	0,04	0,20	1,38	1,25	-0,88	5,00	0,40	0,42	-3,64	-3,42	-1,50	-1,20	-1,08	5,00	-2,17	0,56	1,83	0,13	-0,74	0,53
	AMP	-1,25	-6,06	-1,47		-0,77	5,00	-1,60	1,24	0,22	0,61	1,02	0,94	-0,16	5,00	0,53	0,22	-2,00	-2,84	-1,44	-3,53	-2,22	5,00	-2,41	0,36	2,13	0,13	-0,81	0,55
	UMP	-1,11	-5,90	-3,34	2,51	-0,29	5,00	-1,63	1,42	-1,20	-1,47	1,39	1,38	-0,24	5,00	-0,03	0,61	-1,78	-2,35	-1,08	-5,31	-2,32	5,00	-2,57	0,72	1,60	0,33	-0,94	0,57
	GMP	-1,63	-7,61	-2,70	1,71	-0,52	5,00	-2,15	1,55	-0,33	0,02	0,84	1,22	-0,45	5,00	0,26	0,33	-2,25	-2,00	-0,56	-2,58	-0,79	5,00	-1,63	0,41	2,41	0,17	0,52	0,76
	NADPH	1,39	-2,30	-0,31	2,10	-0,36	5,00	0,11	0,77	1,02	2,19	1,52	-0,49	0,34	5,00	0,91	0,46	0,97	0,29	-0,06	0,71	-2,39	5,00	-0,10	0,60	0,81	0,39	-0,20	0,84
Organic acids	Phosphoenolpyruvic acid	-0,78	-3,23	-2,32		-1,73	5,00	-2,01	0,61	-0,75	0,44	1,07	1,02	-0,27	5,00	0,30	0,36	-1,48	-1,15	0,43	-0,63	-0,53	5,00	-0,67	0,33	2,32	0,03	1,35	0,05
	Citric acid;Isocitric acid	-0,34	-5,73	-2,67	0,64	-0,34	5,00	-1,69	1,15	-0,53	-0,40	-0,16	0,31	0,07	5,00	-0,14	0,15	-0,68	0,16	0,55	-0,98	-0,08	5,00	-0,20	0,28	1,55	0,22	1,48	0,24
	Taurine	-0,55	-6,04	-1,78	1,68	-0,19	5,00	-1,38	1,29	0,01	0,17	0,72	1,13	0,10	5,00	0,43	0,21	-1,63	0,08	0,86	0,09	0,17	5,00	-0,08	0,41	1,80	0,21	1,29	0,37
	2-hydroxybutyric acid	-0,86	-0,20	-1,46	0,86	-0,53	5,00	-0,44	0,39	-0,16	-0,68	0,59	0,25	-0,30	5,00	-0,06	0,22	-1,36	-0,72	-0,11	-1,62	-0,50	5,00	-0,86	0,28	0,38	0,42	-0,43	0,39
	Fumaric acid	-1,06	-3,99	-2,51	0,41	-0,16	5,00	-1,46	0,80	-0,29	-0,37	0,96	0,79	0,11	5,00	0,24	0,27	-1,80	-0,59	0,28	-1,11	-0,60	5,00	-0,76	0,34	1,70	0,08	0,70	0,45
	Urea	-1,21	-6,66	-2,91	0,73	-0,43	5,00	-2,10	1,29	-0,96	-0,67	0,19	0,52	-0,53	5,00	-0,29	0,28	-2,24	-0,90	0,27	-1,92	-0,57	5,00	-1,07	0,46	1,81	0,21	1,02	0,47
	O-phosphoethanolamine	-0,58	-5,92	-2,67	0,35	0,08	5,00	-1,75	1,17	-0,61	-1,09	0,18	0,71	-0,09	5,00	-0,18	0,31	-1,90	-0,74	0,37	-1,50	-0,39	5,00	-0,83	0,40	1,57	0,23	0,92	0,48
	Malic acid	-0,54	-5,28	-2,70	1,06	-0,78	5,00	-1,65	1,09	-0,41	-0,49	0,42	0,57	-0,08	5,00	0,00	0,21	-1,58	-0,74	0,45	-1,64	-0,59	5,00	-0,82	0,38	1,65	0,17	0,83	0,49
	N-acetylputrescine	-0,65	-3,88	-2,08	-0,39	-0,76	5,00	-1,55	0,65	-0,54	-0,68	0,78	-0,25	-0,50	5,00	-0,24	0,26	-1,94	-0,55	0,25	-1,97	-0,77	5,00	-0,99	0,43	1,32	0,10	0,56	0,49
	Lactic acid	-0,88	-4,18	-1,80	0,45	-0,34	5,00	-1,35	0,80	-0,29	-0,41	0,49	0,31	-0,26	5,00	-0,03	0,18	-1,58	-0,51	0,14	-1,36	-0,60	5,00	-0,78	0,31	1,32	0,15	0,57	0,52
	2-hydroxyglutaric acid	-0,63	-4,92	-2,19	0,74	-0,67	5,00	-1,53	0,96	-0,23	-0,45	0,52	0,72	0,29	5,00	0,17	0,22	-1,95	-1,10	0,44	-1,86	-0,55	5,00	-1,00	0,44	1,70	0,12	0,53	0,63
	Hypotaurine	-0,60	-3,44	-2,09	1,36	-0,55	5,00	-1,06	0,81	-0,96	0,03	-0,24	0,37	0,04	5,00	-0,15	0,22	-1,76	-1,01	0,22	-1,11	-0,24	5,00	-0,78	0,35	0,91	0,31	0,28	0,76
	2-oxoglutaric acid	-0,74	-3,52	-2,34	0,52	-0,84	5,00	-1,38	0,70	-0,52	-0,50	-0,24	0,05	-0,39	5,00	-0,32	0,10	-1,71	-1,37	-0,22	-1,81	-0,65	5,00	-1,15	0,31	1,06	0,17	0,23	0,77
	Pyruvic acid;Oxaloacetic acid	-1,03	-2,91	-2,40	0,32	0,30	5,00	-1,14	0,67	-0,56	-0,62	0,39	0,25	-0,38	5,00	-0,18	0,21	-2,10	-0,60	0,12	-1,13	-0,87	5,00	-0,92	0,36	0,96	0,21	0,23	0,77
	Succinic acid	-0,64	-3,27	-1,65	0,83	-0,52	5,00	-1,05	0,68	-0,19	-0,25	0,09	0,07	-0,25	5,00	-0,11	0,08	-1,63	-0,89	-0,59	-1,62	-0,85	5,00	-1,12	0,21	0,94	0,21	-0,07	0,93

Table S1: Metabolite changes in striatum cells from control and PD mouse models (GS-PD or ai-PD). N: number of mice/groups detected for each metabolite; Average: Average log2area; SEM: standard error of the mean; Log2FC: Log2 fold change between two groups and statistical analysis was performed by obtaining *p* values (GS-PD vs control, first column Log2FC; ai-PD vs control second column Log2FC). PCae (phosphatidylcholine); PCee (phosphatidylethanolamine).

Metabolites		Control								GS-PD							ai-PD								GS-PD vs Control		ai-PD vs Control	
Class	Name	Control 1	Control 2	Control 3	Control 4	Control 5	N	AVERAGE	SEM	GS-PD 1	GS-PD 2	GS-PD 3	GS-PD 4	N	AVERAGE	SEM	ai-PD 1	ai-PD 2	ai-PD 3	ai-PD 4	ai-PD 5	N	AVERAGE	SEM	log2FC	TTEST	log2FC	TTEST
Amino acids	3-methylhistidine	1,35	3,99	4,39	1,24	1,82	5	2,56	0,68	3,61	1,21	0,99	1,92	4	1,93	0,59	1,26	4,61	1,67	4,08	0,27	5	2,38	0,84	-0,62	0,522	-0,18	0,872
	4-guanidinobutanoic acid	-0,62	4,42	6,39	-1,73	0,17	5	1,73	1,56	4,36	0,06	-0,88	1,89	4	1,36	1,16	0,75	6,29	-1,84	4,26	-1,09	5	1,67	1,56	-0,37	0,861	-0,05	0,981
	4-hydroxyproline	-0,86	-0,58	-0,52	-1,14	-0,22	5	-0,66	0,16	-1,46	-1,26	-0,34	1,07	4	-0,50	0,58	-0,02	0,69	-1,46	-2,09	-1,15	5	-0,80	0,50	0,17	0,764	-0,14	0,797
	5-aminovaleric acid	-0,67	-0,67	-0,34	-0,49	0,36	5	-0,36	0,19	-1,09	-0,81	-0,28	0,80	4	-0,35	0,42	-0,54	-0,04	-0,46	-0,90	-0,35	5	-0,46	0,14	0,01	0,973	-0,10	0,696
	5-oxoproline	-0,55	-0,63	-0,58	-0,57	0,04	5	-0,46	0,13	-0,95	-0,65	-0,22	-0,35	4	-0,54	0,16	-0,40	-0,16	-0,76	-0,74	-0,38	5	-0,49	0,11	-0,09	0,676	-0,03	0,862
	Alanine	-0,23	-0,36	-0,80	-0,16	0,03	5	-0,30	0,14	-0,79	-0,50	0,09	0,10	4	-0,27	0,22	-0,38	-0,32	-0,45	-0,60	-0,46	5	-0,44	0,05	0,03	0,910	-0,14	0,376
	Arginine	1,96	3,97	4,43	1,87	1,08	5	2,66	0,65	3,72	1,12	1,23	0,95	4	1,76	0,66	1,51	3,94	2,32	4,25	0,19	5	2,44	0,76	-0,91	0,365	-0,22	0,829
	Asparagine	0,05	-0,13	-0,27	-0,23	0,59	5	0,00	0,16	-1,07	-1,06	0,17	0,45	4	-0,38	0,40	-0,26	0,06	-0,68	-0,80	-1,01	5	-0,54	0,19	-0,38	0,366	-0,54	0,060
	Aspartic acid	-0,62	-0,60	-0,84	-0,44	0,46	5	-0,41	0,23	-0,96	-0,32	-0,09	0,80	4	-0,14	0,37	-0,28	0,24	-0,62	-1,19	-0,10	5	-0,39	0,24	0,27	0,538	0,02	0,959
	Beta-alanine	1,10	-0,01	-0,13	0,11	0,09	5	0,23	0,22	0,08	-0,30	-0,81	0,04	4	-0,25	0,21	0,26	-0,36	1,00	1,47	0,21	5	0,52	0,32	-0,48	0,167	0,28	0,489
	Betaine/Valine	2,34	2,86	4,00	2,16	2,59	5	2,79	0,33	2,85	1,25	1,73	2,41	4	2,06	0,35	2,06	3,68	2,00	2,64	0,11	5	2,10	0,58	-0,73	0,175	-0,69	0,327
	Citrulline	-0,07	-0,51	-0,39	-0,44	-0,01	5	-0,28	0,10	-1,04	-0,76	0,05	0,17	4	-0,39	0,30	0,13	-0,09	-0,55	-0,49	-0,90	5	-0,38	0,18	-0,11	0,706	-0,10	0,644
	Creatine	0,68	-0,07	-0,36	-0,03	0,67	5	0,18	0,21	-0,64	0,05	0,08	0,67	4	0,04	0,27	0,92	0,23	1,34	0,08	0,83	5	0,68	0,23	-0,14	0,691	0,50	0,148
	Creatinine	1,63	2,50	2,93	1,56	1,48	5	2,02	0,29	2,38	1,09	1,18	1,93	4	1,64	0,31	1,63	3,68	2,48	3,17	0,78	5	2,35	0,52	-0,37	0,413	0,33	0,595
	Cystine;Cysteine	0,66	-0,57	-0,63	-0,57	0,88	5	-0,04	0,33	-1,64	-0,87	-0,34	0,18	4	-0,67	0,39	0,75	0,15	0,07	-0,64	-0,10	5	0,05	0,22	-0,62	0,263	0,09	0,823
	Dimethylarginine	2,38	2,99	3,49	2,53	2,23	5	2,73	0,23	2,86	1,63	1,30	2,50	4	2,07	0,37	1,78	4,11	2,96	3,70	0,39	5	2,58	0,68	-0,65	0,158	-0,14	0,848
	Dimethylglycine	2,20	2,90	3,72	2,38	1,76	5	2,59	0,34	3,15	1,37	2,20	2,20	4	2,23	0,36	1,32	4,15	1,63	3,48	-0,04	5	2,11	0,76	-0,36	0,488	-0,49	0,574
	GABA	0,34	-0,54	-0,49	-1,00	-0,31	5	-0,40	0,22	-1,31	-1,08	-0,41	0,82	4	-0,50	0,48	0,00	0,46	-0,55	0,08	-0,73	5	-0,15	0,22	-0,10	0,851	0,25	0,435
	Glutamic acid	-0,59	-0,27	0,05	-0,77	0,99	5	-0,12	0,31	-1,45	-0,88	-0,46	1,37	4	-0,35	0,61	-0,29	0,31	-1,27	-1,18	-0,15	5	-0,52	0,31	-0,24	0,724	-0,40	0,389
	Glutamine	-0,22	0,39	0,45	-0,07	1,14	5	0,34	0,24	0,45	-0,06	1,26	1,01	4	0,66	0,30	0,55	0,81	0,24	0,75	0,11	5	0,49	0,14	0,32	0,414	0,15	0,591
	Glycine	0,59	-0,25	-0,25	-0,36	0,06	5	-0,04	0,17	-0,22	-0,33	-0,40	-0,23	4	-0,30	0,04	0,25	-0,09	0,16	0,33	-0,07	5	0,12	0,08	-0,25	0,246	0,16	0,435
	Histidine	0,23	0,11	-0,15	-0,31	0,33	5	0,04	0,12	-0,08	-0,97	0,52	0,75	4	0,05	0,38	0,05	0,30	-0,50	0,24	-0,27	5	-0,04	0,15	0,01	0,975	-0,08	0,696
	Homoserine	0,10	-0,60	-0,61	-0,48	0,08	5	-0,30	0,16	-1,72	-0,42	-0,19	-1,06	4	-0,85	0,34	-0,53	-1,78	-0,10	-0,58	-1,32	5	-0,86	0,30	-0,54	0,167	-0,56	0,142
	Isoleucine	-0,14	-0,46	-0,89	-0,84	-0,32	5	-0,53	0,15	-1,30	-0,33	0,46	-0,51	4	-0,42	0,36	-0,10	-1,30	-0,09	0,12	-1,07	5	-0,49	0,29	0,11	0,767	0,04	0,904
	Leucine	-0,17	-0,60	-0,73	-0,82	-0,39	5	-0,54	0,12	-1,38	-0,78	0,04	-0,58	4	-0,68	0,29	0,01	-1,09	-0,28	-0,22	-1,17	5	-0,55	0,24	-0,14	0,653	-0,01	0,971
	Lysine	0,03	-0,20	-0,20	-0,63	0,22	5	-0,16	0,14	-0,30	-1,19	0,35	0,84	4	-0,07	0,44	-0,06	0,07	-0,24	0,13	-0,83	5	-0,19	0,17	0,08	0,849	-0,03	0,898
	Methionine	0,25	-0,44	-0,56	-0,27	0,40	5	-0,12	0,19	-1,72	-1,03	-0,30	0,02	4	-0,76	0,39	-0,72	-0,17	-0,16	-1,02	-1,26	5	-0,66	0,22	-0,63	0,161	-0,54	0,102
	N-acetylglutamic acid	2,63	4,01	0,54	2,74	2,89	5	2,56	0,56	4,71	1,85	2,35	3,24	4	3,04	0,63	3,23	6,05	2,06	4,41	0,61	5	3,27	0,94	0,48	0,589	0,71	0,534
	N1-acetylspermidine	0,13	-0,91	-0,07	-0,17	0,76	5	-0,05	0,27	0,08	-1,32	-0,36	-0,56	4	-0,54	0,29	0,17	0,28	0,57	-0,33	-0,02	5	0,13	0,15	-0,49	0,259	0,19	0,565
	N6-acetyllysine	2,12	3,05	3,73	1,75	1,76	5	2,48	0,39	2,71	1,98	1,37	1,76	4	1,95	0,28	1,45	4,26	2,18	3,61	0,24	5	2,35	0,72	-0,53	0,333	-0,13	0,877
	Ornithine	0,38	-0,39	-0,27	-0,42	0,18	5	-0,10	0,16	-0,73	-0,98	-0,15	0,58	4	-0,32	0,35	0,20	-0,25	-0,30	-0,13	-0,82	5	-0,26	0,16	-0,22	0,560	-0,15	0,524
	Pantothenic acid	-0,98	-0,44	-0,11	-0,43	0,39	5	-0,31	0,22	-0,89	-1,16	-0,32	1,09	4	-0,32	0,50	0,21	-0,25	-0,69	-1,13	-0,43	5	-0,46	0,22	0,00	0,994	-0,15	0,653
	Phenylalanine	-0,12	-0,43	-0,71	-0,43	0,35	5	-0,27	0,18	-1,08	-1,07	0,02	0,31	4	-0,46	0,36	-0,60	0,01	-0,52	-0,78	-0,79	5	-0,54	0,15	-0,19	0,636	-0,27	0,281
	Proline	-0,71	-0,86	-1,52	-0,85	-0,39	5	-0,86	0,18	-1,78	-0,94	0,44	0,83	4	-0,37	0,61	0,14	-1,40	-1,47	-1,15	-2,06	5	-1,19	0,36	0,50	0,412	-0,33	0,447
	Proline betaine	2,46	2,66	3,95	2,55	3,27	5	2,98	0,28	3,45	2,01	2,66	3,33	4	2,86	0,33	2,10	4,12	2,18	2,81	0,03	5	2,25	0,66	-0,12	0,796	-0,73	0,339
	Serine	0,14	-0,40	-0,34	-0,36	0,42	5	-0,11	0,16	-1,45	-0,24	0,12	-0,72	4	-0,57	0,34	-0,04	-0,97	-0,12	-0,39	-1,22	5	-0,55	0,23	-0,46	0,229	-0,44	0,164
	Threonine	0,04	-0,14	-0,55	-0,18	0,41	5	-0,08	0,16	-1,01	-0,87	0,00	-0,21	4	-0,52	0,25	-0,57	0,06	-0,34	-0,75	-0,73	5	-0,47	0,15	-0,44	0,165	-0,38	0,119
	Trimethyl-lysine	1,79	4,12	4,47	1,39	2,09	5	2,77	0,63	4,03	1,48	1,18	1,81	4	2,12	0,65	1,49	4,49	2,33	4,53	0,20	5	2,61	0,85	-0,65	0,503	-0,16	0,882
	Tryptophan	0,50	-0,55	-1,17	-0,49	0,24	5	-0,29	0,30	-1,82	-0,89	0,06	0,24	4	-0,60	0,47	0,04	-0,48	0,18	-0,52	-1,26	5	-0,41	0,25	-0,31	0,582	-0,11	0,779
	Tyrosine	0,13	-0,17	-0,47	-0,26	0,24	5	-0,11	0,13	-0,98	-1,11	0,06	0,46	4	-0,39	0,39	-0,43	-0,09	-0,31	-0,50	-1,07	5	-0,48	0,16	-0,28	0,465	-0,37	0,113
	Valine	0,09	-0,34	-0,99	-0,83	0,22	5	-0,37	0,24	-0,77	-0,84	-0,23	0,00	4	-0,46	0,21	-0,64	-0,62	0,07	-0,45	-0,69	5	-0,46	0,14	-0,09	0,788	-0,09	0,744
Bases	Adenine	0,06	-0,23	-0,09	-0,66	0,30	5	-0,12	0,16	-0,29	-0,36	-0,05	0,22	4	-0,12	0,13	0,23	0,04	-0,17	-0,18	-0,06	5	-0,03	0,08	0,01	0,979	0,10	0,602
	Cytosine	0,03	-0,14	0,37	-0,48	0,29	5	0,01	0,15	-0,72	0,06	-0,30	0,98	4	0,00	0,36	0,22	0,29	-0,05	0,12	-0,71	5	-0,03	0,18	-0,01	0,981	-0,04	0,870
	Guanine	0,05	-1,10	-0,26	0,15	-0,23	5	-0,28	0,22	-0,90	-1,23	-0,19	0,83	4	-0,37	0,46	0,04	-0,08	-0,27	-1,20	-2,94	5	-0,89	0,56	-0,09	0,849	-0,61	0,337
	Hypoxanthine	0,12	-0,24	-0,34	0,06	0,57	5	0,03	0,16	-0,79	-1,10	-0,36	0,45	4	-0,45	0,34	-0,58	-0,51	0,45	-0,23	-0,54	5	-0,28	0,19	-0,48	0,204	-0,32	0,243
	Thymine	-0,63	-0,32	1,26	0,78	0,69	5	0,36	0,36	-0,89	-0,24	-0,02	1,17	4	0,01	0,43	0,79	0,45	-0,55	-0,35	-0,20	5	0,03	0,25	-0,35	0,545	-0,33	0,474
	Uracil	-0,65	0,68	0,15	-0,35	0,12	5	-0,01	0,23	-0,12	-1,27	-0,58	0,27	4	-0,43	0,33	-0,17	-0,11	-0,48	0,01	-0,14	5	-0,18	0,08	-0,41	0,322	-0,17	0,510
	Uric acid	-1,39	-0,46	-1,58	-0,45	-0,81	5	-0,94	0,23	-0,92	-1,32	-0,02	-1,15	4	-0,85	0,29	0,67	-0,69	-0,84	-0,64	-0,64	5	-0,43	0,28	0,08	0,825	0,51	0,196
	Xanthine	0,09	-0,34	-0,62	0,00	0,47	5	-0,08	0,19	-0,66	-1,28	-0,22	-0,05	4	-0,55	0,27	-0,56	-0,58	0,36	-0,38	-0,59	5	-0,35	0,18	-0,47	0,184	-0,27	0,334
Bile acids	CA	4,03	4,59	1,28	-0,38	-0,18	5	1,87	1,04	3,02	0,29	-2,35	0,90	4	0,47	1,11	-0,89	0,98	4,81	7,51	-0,42	5	2,40	1,62	-1,40	0,389	0,53	0,791
	Cholesterol	-0,16	0,06	-0,22	-0,36	0,09	5	-0,12	0,08	-0,31	-0,49	-0,27	0,06	4	-0,25	0,11	-0,03	0,08	-0,11	0,01	-0,19	5	-0,05	0,05	-0,13	0,374	0,07	0,478
	G-CA	4,02	5,20	5,23	2,59	2,11	5	3,83	0,65	4,93	2,20	2,09	3,67	4	3,22	0,67	1,92	5,08	4,50	6,83	1,28	5	3,92	1,03	-0,60	0,542	0,09	0,940
	T-CA	3,48	5,15	3,83	1,06	1,14	5	2,93	0,80	4,07	1,47	0,84	3,14	4	2,38	0,74	0,87	3,59	4,42	5,93	0,60	5	3,08	1,03	-0,55	0,637	0,15	0,913
	T-CDCA	3,17	4,06	3,01	2,68	2,81	5	3,14	0,24	2,77	1,77	1,84	1,96	4	2,09	0,23	1,85	3,16	3,83	4,50	1,62	5	2,99	0,56	-1,06	0,017	-0,16	0,804
	T-DCA	3,17	4,31	3,75	2,68	2,81	5	3,34	0,30	3,96	1,77	1,84	1,96	4	2,38	0,53	1,85	5,16	3,83	5,43	1,62	5	3,58	0,80	-0,96	0,140	0,24	0,791
	T-UDCA/T-HDCA	4,06	5,73	4,75	2,42	2,76	5	3,94	0,62	3,84	2,44	1,02	2,27	4	2,39	0,58	1,30	4,69	4,77	7,38	1,29	5	3,89	1,16	-1,55	0,115	-0,06	0,965
Carbohydrates	Arabinose	0,08	-0,20	0,02	-0,80	0,09	5	-0,16	0,17	-0,63	-0,84	-0,37	0,17	4	-0,42	0,22	-0,03	0,41	-0,55	-0,45	-0,47	5	-0,22	0,18	-0,26	0,365	-0,06	0,823
	Arabitol	-0,94	-0,16	-0,56	-1,05	-0,21	5	-0,59	0,18	-0,56	-1,12	-0,40	-0,21	4	-0,57	0,20	-0,12	-0,04	-1,00	-0,66	-0,72	5	-0,51	0,18	0,02	0,955	0,08	0,770
	Erythritol	-0,60	-0,08	-0,43	-0,19	0,14	5	-0,23	0,13	-0,72	-0,71	-0,09	-0,04	4	-0,39	0,19	-0,32	-0,27	-0,67	-0,83	-0,53	5	-0,52	0,10	-0,16	0,498	-0,29	0,118
	Fructose	-0,81	0,04	-0,30	0,35	0,68	5	-0,01	0,26	-0,51	-0,62	-0,41	-0,27	4	-0,45	0,07	-1,00	-0,09	0,03	-0,13	-0,38	5	-0,32	0,18	-0,45	0,182	-0,31	0,360
	Galactitol;Sorbitol;Mannitol	-0,09	0,00	-0,13	-0,09	0,05	5	-0,05	0,03	-0,14	-0,30	-0,11	-0,10	4	-0,16	0,05	-0,10	-0,04	-0,13	-0,10	-0,13	5	-0,10	0,02	-0,11	0,080	-0,05	0,233
	Galactose;Glucose;Mannose	-0,08	-0,05	-0,12	-0,10	-0,03	5	-0,08	0,02	-0,17	-0,25	-0,14	-0,12	4	-0,17	0,03	-0,13	-0,05	-0,13	-0,15	-0,16	5	-0,13	0,02	-0,09	0,022	-0,05	0,096
	Glucose-6-phosphate	1,64	4,68	5,43	2,71	2,28	5	3,35	0,73	5,69	1,82	1,42	3,00	4	2,98	0,96	1,62	6,23	2,70	5,52	1,21	5	3,46	1,02	-0,36	0,767	0,11	0,933
	Fructose-6-phosphate	-0,64	-4,64	-2,70	0,14	0,03	5	-1,56	0,92	-1,13	-0,83	-1,31	1,39	4	-0,47	0,63	-1,55	-0,17	0,39	-2,08	-0,80	5	-0,84	0,45	1,09	0,386	0,72	0,502
	Glucuronic acid;Galacturonic acid	3,40	5,28	5,80	3,13	3,21	5	4,16	0,57	4,47	1,74	1,94	2,61	4	2,69	0,62	3,06	5,77	2,77	5,55	0,50	5	3,53	0,98	-1,47	0,125	-0,63	0,590
	Glyceric acid	-0,63	-0,44	-0,64	-0,73	-0,38	5	-0,57	0,07	-0,73	-0,95	-0,64	-0,50	4	-0,70	0,10	-0,31	-0,45	-0,80	-0,63	-0,40	5	-0,52	0,09	-0,14	0,266	0,05	0,668
	Glycerol	-0,25	-0,51	-1,13	-0,22	-0,30	5	-0,48	0,17	-1,00	-0,75	-0,53	-1,04	4	-0,83	0,12	-1,23	-0,91	-0,43	-1,01	-1,01	5	-0,92	0,13	-0,35	0,155	-0,44	0,076
	Lactose	0,37	-0,21	0,29	-0,48	0,11	5	0,02	0,16	-0,06	-0,62	0,02	0,46	4	-0,05	0,22	0,11	-0,32	-0,31	-0,02	-0,15	5	-0,14	0,08	-0,07	0,807	-0,16	0,411
	Maltose;Trehalose	0,07	0,50	-0,22	0,13	0,41	5	0,18	0,13	-0,68	-1,60	-1,17	-0,76	4	-1,05	0,21	-1,26	-1,19	0,23	-0,14	-1,11	5	-0,69	0,31	-1,23	0,001	-0,87	0,031
	Mannitol	1,85	4,63	4,40	1,25	1,25	5	2,68	0,76	3,90	0,94	0,04	-0,01	4	1,22	0,92	-0,15	3,88	1,33	4,63	0,76	5	2,09	0,92	-1,46	0,257	-0,59	0,636
	N-glycolylneuraminic acid	2,29	4,94	5,63	2,12	2,39	5	3,47	0,75	5,30	1,92	1,61	2,57	4	2,85	0,84	2,20	6,27	2,56	6,02	0,74	5	3,56	1,10	-0,62	0,597	0,09	0,950
	Raffinose	0,57	0,53	-0,31	-1,21	-0,38	5	-0,16	0,33	-0,11	-0,73	-0,61	-0,81	4	-0,56	0,16	0,07	0,55	0,62	0,47	-0,96	5	0,15	0,29	-0,40	0,344	0,31	0,507
	Ribitol	0,07	0,40	-0,22	-0,12	1,08	5	0,24	0,23	-0,97	-1,02	-0,86	-0,89	4	-0,94	0,04	-1,06	-0,57	0,25	-0,49	-0,79	5	-0,53	0,22	-1,18	0,003	-0,78	0,042
	Ribose-5-phosphate;Ribulose-5-phospha	2,43	4,66	4,77	2,82	2,28	5	3,39	0,55	5,31	2,19	2,15	2,50	4	3,04	0,76	1,89	5,53	3,14	5,45	1,06	5	3,41	0,91	-0,35	0,710	0,02	0,985
	Ribose;Ribulose	0,15	-0,16	-0,68	0,11	0,45	5	-0,03	0,19	-0,98	-1,06	-0,74	-1,07	4	-0,96	0,08	-0,98	-0,89	0,02	-0,43	-0,74	5	-0,60	0,18	-0,94	0,004	-0,58	0,059
	Sucrose	0,96	-0,40	-0,15	-0,10	-0,32	5	0,00	0,25	-0,06	-1,00	-0,49	0,19	4	-0,34	0,26	-0,67	-0,87	1,86	-0,35	-0,76	5	-0,16	0,51	-0,34	0,377	-0,16	0,789
	Threonic acid	-0,63	-0,10	-0,22	-0,60	0,24	5	-0,26	0,16	-0,64	-0,55	-0,22	-0,10	4	-0,38	0,13	-0,44	-0,18	-0,74	-0,56	-0,26	5	-0,44	0,10	-0,11	0,615	-0,17	0,389
	Xylitol	0,03	0,11	-0,33	-0,11	0,54	5	0,05	0,14	-0,46	-1,02	-0,77	-0,18	4	-0,61	0,18	-0,43	-0,19	-0,27	-0,16	-0,26	5	-0,26	0,05	-0,65	0,025	-0,31	0,076
	Xylose	1,29	-0,06	-0,70	-0,01	-0,17	5	0,07	0,33	-1,56	-0,67	-0,39	0,04	4	-0,64	0,34	-0,30	0,17	-0,89	-0,41	-1,54	5	-0,59	0,29	-0,72	0,178	-0,67	0,167
Fatty Acyls	Acetylcarnitine	1,75	3,52	3,84	1,57	1,92	5	2,52	0,48	3,09	2,23	2,35	3,10	4	2,69	0,24	2,90	4,57	1,04	3,36	0,50	5	2,47	0,75	0,17	0,776	-0,05	0,960
	AcetylCoA	-1,30	-0,05	-0,19	-1,09	-0,52	5	-0,63	0,24	0,48	0,38	0,91	0,34	4	0,53	0,13	0,89	0,21	-1,68	-0,65	-0,44	5	-0,33	0,43	1,16	0,006	0,30	0,567
	Arachidic acid	1,28	1,93	2,57	1,08	1,37	5	1,64	0,27	1,44	0,97	0,11	0,49	4	0,75	0,29	0,87	2,67	1,12	1,81	1,23	5	1,54	0,32	-0,89	0,061	-0,10	0,813
	Arachidonic acid	0,16	-0,56	-0,99	-0,55	-0,28	5	-0,45	0,19	-1,20	-0,51	-0,50	-0,74	4	-0,74	0,16	-0,69	-0,76	0,06	-0,88	-0,83	5	-0,62	0,17	-0,29	0,297	-0,18	0,512
	Capric acid	0,05	3,18	4,34	0,16	0,37	5	1,62	0,89	3,90	0,07	0,04	0,12	4	1,03	0,96	0,18	4,00	0,02	4,00	0,45	5	1,73	0,93	-0,59	0,670	0,11	0,935
	Caproic acid	-0,10	3,37	3,25	-0,12	-0,13	5	1,25	0,84	3,03	0,03	0,11	-0,05	4	0,78	0,75	-0,08	3,01	-0,24	3,06	0,18	5	1,19	0,76	-0,47	0,695	-0,07	0,954
	Caprylic acid	-0,11	2,44	2,73	0,02	-0,50	5	0,92	0,69	2,49	0,06	-0,26	-0,39	4	0,47	0,68	-0,28	-0,29	0,03	1,48	-0,06	5	0,18	0,33	-0,44	0,666	-0,74	0,362
	Carnitine	2,28	3,62	3,93	2,42	2,60	5	2,97	0,34	2,95	1,37	1,69	2,51	4	2,13	0,36	1,60	3,87	2,46	3,88	0,63	5	2,49	0,64	-0,84	0,135	-0,48	0,523
	Carnitine C18:0	4,53	3,49	3,64	3,30	5,23	5	4,04	0,37	0,96	3,88	1,92	3,49	4	2,56	0,68	4,53	4,55	5,15	2,32	3,60	5	4,03	0,49	-1,48	0,082	-0,01	0,987
	Carnitine C18:1	2,79	3,43	3,31	1,80	3,89	5	3,04	0,36	1,70	1,82	1,72	3,36	4	2,15	0,41	3,51	4,29	3,35	2,72	2,63	5	3,30	0,30	-0,89	0,141	0,26	0,598
	Carnitine C3:0	1,72	3,88	3,51	1,90	2,38	5	2,68	0,43	3,44	0,60	1,94	3,16	4	2,28	0,65	2,57	3,87	2,64	3,77	1,44	5	2,86	0,45	-0,39	0,618	0,18	0,780
	Carnitine C4:0	2,78	3,80	3,60	2,04	2,27	5	2,90	0,35	2,56	1,53	1,87	2,55	4	2,13	0,26	3,56	4,31	1,49	3,57	0,45	5	2,67	0,73	-0,77	0,136	-0,22	0,792
	Carnitine C4:0(OH)	2,45	3,68	4,15	2,42	3,16	5	3,17	0,34	3,83	1,94	2,16	2,40	4	2,58	0,42	2,22	4,05	2,69	4,08	0,70	5	2,75	0,63	-0,59	0,308	-0,42	0,573
	Carnitine C6:0	2,89	3,13	2,72	1,89	1,86	5	2,50	0,26	1,09	0,74	1,23	2,32	4	1,34	0,34	4,23	3,42	1,65	2,42	0,11	5	2,36	0,71	-1,16	0,029	-0,13	0,864
	Docosahexaenoic acid	3,37	3,08	2,74	2,81	3,39	5	3,08	0,14	1,68	1,78	0,21	1,51	4	1,29	0,37	1,96	3,52	4,12	3,13	2,43	5	3,03	0,38	-1,78	0,002	-0,05	0,911
	Docosanedioic acid	0,34	0,81	1,83	-0,04	-0,05	5	0,58	0,35	0,90	0,16	-0,21	-0,05	4	0,20	0,24	-0,05	1,41	0,19	0,75	-0,07	5	0,45	0,28	-0,38	0,427	-0,13	0,775
	Docosapentaenoic acid	3,74	3,48	2,91	3,39	3,87	5	3,48	0,17	1,18	2,26	0,11	1,23	4	1,19	0,44	2,16	3,41	4,23	2,92	2,54	5	3,05	0,36	-2,28	0,001	-0,43	0,316
	Docosatetraenoic acid	3,26	2,56	1,93	2,63	3,19	5	2,71	0,24	0,33	2,35	0,27	1,63	4	1,14	0,51	2,32	3,36	4,43	2,50	2,53	5	3,03	0,39	-1,57	0,020	0,31	0,515
	Docosatrienoic acid	3,14	3,50	3,03	2,70	4,24	5	3,32	0,26	2,60	3,01	1,41	3,25	4	2,57	0,41	2,85	5,06	4,50	3,58	3,21	5	3,84	0,41	-0,75	0,150	0,52	0,318
	Docosenoic acid	2,66	3,19	3,67	2,76	3,32	5	3,12	0,19	2,42	2,67	0,74	1,74	4	1,89	0,43	2,03	4,35	2,20	2,57	2,47	5	2,73	0,42	-1,23	0,025	-0,39	0,414
	Dodecanedioic acid	0,02	2,71	2,82	0,05	0,02	5	1,12	0,67	2,76	-0,08	-0,13	-0,09	4	0,61	0,72	0,03	2,26	-0,21	2,64	0,33	5	1,01	0,60	-0,51	0,620	-0,11	0,901
	Dodecenedioic acid	-0,79	4,18	3,70	-0,30	-1	4	1,70	1,30	4,38	-0,44	0,52	0,49	4	1,24	1,07	0,20	4,12	-0,32	4,09	0,80	5	1,78	0,97	-0,46	0,792	0,08	0,962
	Dodecenoic acid	0,23	4,60	5,26	0,58	1,13	5	2,36	1,07	5,05	0,18	0,53	0,38	4	1,54	1,17	0,28	5,38	1,00	5,10	1,10	5	2,57	1,10	-0,83	0,619	0,21	0,893
	Eicosadienoic acid	3,13	2,60	2,74	2,46	3,59	5	2,90	0,21	1,42	2,33	0,57	1,99	4	1,58	0,38	1,76	3,53	4,15	2,79	2,31	5	2,91	0,43	-1,33	0,014	0,00	0,996
	Eicosapentaenoic acid	2,90	3,08	3,07	2,72	2,87	5	2,93	0,07	1,78	1,50	0,23	0,94	4	1,11	0,34	1,64	3,28	2,84	2,38	1,71	5	2,37	0,32	-1,82	0,001	-0,56	0,124
	Eicosatrienoic acid	3,02	3,35	2,93	2,80	3,65	5	3,15	0,15	2,07	2,04	0,39	1,82	4	1,58	0,40	1,97	3,76	4,01	2,98	2,43	5	3,03	0,39	-1,57	0,005	-0,12	0,781
	Eicosenoic acid	2,83	2,74	2,65	2,45	3,48	5	2,83	0,17	1,53	2,57	0,93	1,96	4	1,75	0,35	1,78	3,66	3,50	2,39	2,29	5	2,72	0,37	-1,08	0,020	-0,11	0,799
	Glutarylcarnitine	3,20	3,90	4,52	3,33	3,06	5	3,60	0,27	3,48	2,18	2,29	3,82	4	2,94	0,42	2,45	4,92	3,03	4,58	1,28	5	3,25	0,68	-0,66	0,209	-0,35	0,644
	Hexadecadienoic acid	2,33	3,70	3,59	2,38	2,98	5	3,00	0,29	2,78	1,33	0,75	1,54	4	1,60	0,43	1,61	4,43	3,07	3,59	2,20	5	2,98	0,50	-1,40	0,026	-0,02	0,975
	Hexadecanedioic acid	-0,04	2,20	2,74	0,00	-0,07	5	0,97	0,62	2,30	-0,05	0,24	0,07	4	0,64	0,56	0,03	2,96	0,01	2,08	0,03	5	1,02	0,63	-0,33	0,713	0,06	0,949
	Lauric acid	0,00	2,55	3,70	0,21	0,14	5	1,32	0,76	3,10	0,12	-0,11	-0,13	4	0,74	0,79	0,15	3,60	0,04	2,98	0,32	5	1,42	0,77	-0,57	0,620	0,10	0,930
	Linoleic acid	0,28	-0,02	-0,56	-0,25	0,42	5	-0,03	0,18	-0,56	-0,23	-0,03	-0,08	4	-0,23	0,12	-0,86	-0,78	0,21	-0,24	-0,60	5	-0,45	0,20	-0,20	0,407	-0,42	0,150
	Linolenic acid	3,69	3,45	3,38	3,27	3,50	5	3,46	0,07	1,78	1,99	-0,13	1,27	4	1,23	0,48	1,98	3,88	4,08	3,53	2,45	5	3,18	0,41	-2,23	0,001	-0,27	0,530
	Malonylcarnitine	2,02	4,28	3,41	2,51	2,53	5	2,95	0,40	3,25	1,69	1,76	2,17	4	2,22	0,36	1,40	3,26	1,53	3,38	0,56	5	2,02	0,55	-0,73	0,230	-0,92	0,214
	Margaric acid	0,29	-0,55	-0,19	-0,07	-0,10	5	-0,12	0,13	-0,52	-0,11	-0,26	-0,05	4	-0,24	0,10	-0,21	-0,24	0,03	-0,32	-0,33	5	-0,21	0,06	-0,11	0,538	-0,09	0,552
	Myristic acid	1,66	2,33	2,87	1,27	1,55	5	1,94	0,29	1,85	1,19	0,23	0,64	4	0,97	0,35	1,07	3,14	1,61	2,26	1,35	5	1,89	0,37	-0,96	0,071	-0,05	0,918
	Myristoleic acid	0,68	1,90	2,52	0,47	0,98	5	1,31	0,39	1,74	0,42	0,13	0,45	4	0,69	0,36	0,25	2,53	0,91	1,71	0,39	5	1,16	0,43	-0,62	0,289	-0,15	0,805
	Nonadecanoic acid	1,09	2,23	2,88	1,08	1,32	5	1,72	0,36	2,01	0,51	-0,10	0,56	4	0,74	0,45	0,93	3,08	1,50	2,58	1,18	5	1,85	0,42	-0,98	0,127	0,13	0,815
	Nonadecenoic acid	2,71	2,99	3,19	2,24	3,22	5	2,87	0,18	1,85	1,39	0,34	1,38	4	1,24	0,32	1,57	3,90	3,69	3,63	2,19	5	2,99	0,47	-1,63	0,002	0,12	0,813
	Nonanoic acid	0,05	2,86	3,41	0,02	0,00	5	1,27	0,77	3,00	0,05	-0,10	-0,02	4	0,73	0,76	-0,09	2,30	0,08	2,96	0,07	5	1,07	0,65	-0,54	0,638	-0,20	0,845
	Oleic acid;Elaidic acid	0,23	-0,42	-0,76	-0,34	0,37	5	-0,19	0,21	-0,34	-0,24	-0,04	0,28	4	-0,08	0,14	-1,18	-0,99	0,38	-0,14	-0,63	5	-0,51	0,28	0,10	0,716	-0,33	0,383
	Palmitic acid	0,26	-0,46	0,08	0,05	0,04	5	-0,01	0,12	-0,46	-0,13	-0,44	0,03	4	-0,25	0,12	-0,08	0,10	-0,07	-0,23	-0,41	5	-0,13	0,09	-0,24	0,201	-0,13	0,412
	Palmitoleic acid	0,28	0,01	-0,19	0,10	0,49	5	0,14	0,12	-0,81	-0,30	-0,42	0,26	4	-0,32	0,22	-0,53	-0,19	0,06	-0,43	-0,41	5	-0,30	0,11	-0,45	0,094	-0,43	0,024
	Pentadecanoic acid	1,97	2,46	3,10	1,26	1,41	5	2,04	0,34	2,00	0,97	0,25	0,51	4	0,93	0,39	1,13	3,23	1,83	2,57	1,29	5	2,01	0,40	-1,10	0,069	-0,03	0,960
	Sebacic acid	-0,01	3,03	2,78	-0,08	0,10	5	1,16	0,71	-0,27	0,03	-0,15	-1,01	4	-0,35	0,23	-0,10	0,31	0,01	1,51	0,28	5	0,40	0,29	-1,51	0,112	-0,76	0,350
	Stearic acid	1,02	1,99	2,55	0,86	1,14	5	1,51	0,32	1,69	0,71	-0,04	0,57	4	0,73	0,36	0,79	2,65	1,17	2,11	1,19	5	1,58	0,34	-0,78	0,151	0,07	0,893
	Stearidonic acid	3,49	7,00	7,04	3,73	4,04	5	5,06	0,81	5,63	1,55	0,10	1,22	4	2,13	1,21	2,66	6,87	3,20	6,84	2,99	5	4,51	0,96	-2,93	0,075	-0,55	0,674
	Tetracosenoic acid	3,98	4,36	5,01	4,11	4,81	5	4,45	0,20	3,99	3,65	1,28	3,00	4	2,98	0,60	3,60	5,84	4,48	4,41	3,93	5	4,45	0,38	-1,47	0,038	0,00	0,998
	Tetradecadienoic acid	NA	2,47	NA	1,04	1,74	3	1,75	0,41	2,45	0,95	0,17	0,55	4	1,03	0,50	0,40	NA	2,74	2,98	1,10	4	1,81	0,63	-0,72	0,342	0,06	0,947
	Tetradecanedioic acid	0,12	2,04	2,82	-0,09	-0,03	5	0,97	0,61	2,42	0,12	-0,21	-0,10	4	0,56	0,62	-0,40	2,63	-0,09	2,11	-0,04	5	0,84	0,63	-0,41	0,655	-0,13	0,887
	Undecanedioic acid	0,07	2,87	2,73	0,03	0,14	5	1,17	0,67	2,83	0,02	0,02	0,32	4	0,80	0,68	0,02	2,68	0,09	2,55	0,20	5	1,11	0,62	-0,37	0,712	-0,06	0,947
	Undecanoic acid	-0,09	3,26	3,84	0,02	0,12	5	1,43	0,87	3,45	-0,03	-0,17	0,06	4	0,83	0,87	0,02	3,57	-0,07	3,22	0,34	5	1,42	0,81	-0,60	0,645	-0,01	0,991
Lipids	Glycerol-3-phosphate	0,09	-0,84	-0,46	-0,34	-0,55	5	-0,42	0,15	-0,39	0,08	0,18	-0,59	4	-0,18	0,19	0,39	-0,55	-0,61	-0,47	-1,26	5	-0,50	0,26	0,24	0,343	-0,08	0,804
	Glycerophosphorylcholine	2,47	4,02	4,41	1,34	1,66	5	2,78	0,62	3,36	2,34	0,44	1,06	4	1,80	0,65	2,38	3,92	2,58	4,68	-0,23	5	2,67	0,84	-0,98	0,315	-0,11	0,916
	PCae(14:0)	3,88	2,48	2,67	3,28	3,68	5	3,20	0,27	2,12	3,45	1,39	2,28	4	2,31	0,43	3,33	3,91	4,32	2,74	2,63	5	3,39	0,33	-0,89	0,111	0,19	0,672
	PCae(16:0)	2,83	3,83	3,59	2,68	3,35	5	3,26	0,22	2,74	2,24	0,82	1,76	4	1,89	0,41	3,13	4,92	3,52	3,85	2,85	5	3,65	0,36	-1,37	0,017	0,40	0,372
	PCae(16:1)	3,05	3,08	3,14	2,93	3,54	5	3,15	0,10	2,65	2,66	0,85	2,12	4	2,07	0,43	2,70	4,28	3,62	3,35	2,72	5	3,33	0,30	-1,08	0,028	0,19	0,571
	PCae(17:0)	3,05	2,68	2,83	2,53	3,17	5	2,85	0,12	1,79	2,03	0,30	1,51	4	1,41	0,38	3,03	4,12	4,32	3,43	2,68	5	3,51	0,31	-1,44	0,005	0,66	0,083
	PCae(18:0)	2,47	3,49	2,92	2,08	2,98	5	2,79	0,24	2,41	1,97	0,70	1,53	4	1,65	0,36	3,05	4,50	3,54	3,67	2,88	5	3,53	0,29	-1,14	0,030	0,74	0,083
	PCae(18:1)	2,55	3,31	3,14	2,28	3,32	5	2,92	0,21	3,15	2,49	0,87	2,16	4	2,17	0,48	2,85	4,69	3,55	3,81	2,82	5	3,54	0,35	-0,75	0,165	0,62	0,163
	PCae(18:2)	3,00	3,14	3,17	2,68	3,50	5	3,10	0,13	2,72	2,43	0,71	1,78	4	1,91	0,44	3,00	4,49	3,89	3,99	2,92	5	3,66	0,30	-1,19	0,025	0,56	0,128
	PCae(20:1)	3,00	4,07	NA	2,93	3,98	4	3,49	0,31	4,01	3,37	1,37	3,22	4	2,99	0,57	3,74	5,66	4,12	4,25	3,51	5	4,25	0,37	-0,50	0,467	0,76	0,173
	PCae(20:3)	2,67	3,56	2,87	2,30	3,57	5	2,99	0,25	2,97	2,41	1,15	2,07	4	2,15	0,38	2,81	NA	3,72	3,83	3,16	4	3,38	0,24	-0,84	0,096	0,39	0,310
	PCae(20:4)	2,53	3,07	2,47	2,08	3,02	5	2,63	0,18	2,39	2,01	0,75	1,54	4	1,67	0,35	2,56	4,27	4,00	3,99	3,19	5	3,60	0,32	-0,96	0,037	0,97	0,030
	PCae(20:5)	3,40	1,71	2,57	3,02	3,23	5	2,79	0,30	0,92	3,72	0,90	2,18	4	1,93	0,67	3,21	2,60	4,47	1,85	2,38	5	2,90	0,45	-0,86	0,248	0,11	0,838
	PCae(22:5)	3,87	9,61	8,52	4,58	6,15	5	6,55	1,11	8,68	3,11	2,03	4,46	4	4,57	1,46	4,96	NA	4,95	10,33	6,47	4	6,68	1,27	-1,98	0,305	0,13	0,941
	PCae(22:6)	2,96	3,48	2,86	2,66	3,43	5	3,08	0,16	2,49	2,31	0,72	1,18	4	1,67	0,43	2,68	3,95	3,97	3,78	2,93	5	3,46	0,27	-1,40	0,012	0,39	0,261
	PCee(16:0)	3,62	3,89	3,86	2,75	4,28	5	3,68	0,26	1,61	2,41	0,85	2,73	4	1,90	0,42	4,09	5,47	5,12	4,39	3,83	5	4,58	0,31	-1,78	0,007	0,90	0,056
Nucleosides	Adenosine	-0,34	-0,67	-0,21	-0,95	0,28	5	-0,38	0,21	-0,79	0,03	-0,16	2,00	4	0,27	0,60	-0,26	0,30	0,05	-0,05	0,06	5	0,02	0,09	0,65	0,298	0,40	0,118
	Cytidine	0,87	-0,73	-0,07	0,40	0,46	5	0,19	0,27	0,34	0,59	-0,09	0,75	4	0,39	0,18	-2,11	-0,08	-0,23	-0,31	-1,08	5	-0,76	0,38	0,21	0,571	-0,95	0,076
	Guanosine	-0,13	0,03	0,29	-0,40	-0,22	5	-0,09	0,12	0,16	-0,89	0,16	0,38	4	-0,05	0,28	-0,49	-0,43	-0,60	-0,53	-0,20	5	-0,45	0,07	0,04	0,898	-0,36	0,027
	Inosine	-0,05	-0,13	-0,57	-0,24	0,38	5	-0,12	0,15	-0,75	-0,62	-0,18	0,11	4	-0,36	0,20	-0,07	-0,23	-0,17	-0,35	-0,26	5	-0,22	0,05	-0,24	0,366	-0,09	0,574
	S-adenosylmethionine	2,74	2,73	3,18	1,10	2,20	5	2,39	0,36	2,82	2,58	1,23	1,25	4	1,97	0,42	2,26	3,65	2,72	3,38	0,98	5	2,60	0,47	-0,42	0,472	0,21	0,736
	Succinyladenosine	2,80	5,47	5,40	3,77	3,71	5	4,23	0,52	5,11	2,47	1,48	2,05	4	2,78	0,80	2,00	5,76	3,52	5,03	1,14	5	3,49	0,87	-1,45	0,159	-0,74	0,488
	Uridine	2,08	4,91	4,70	2,42	2,15	5	3,25	0,64	4,43	1,06	1,18	1,45	4	2,03	0,80	1,05	4,83	2,58	4,69	0,64	5	2,76	0,88	-1,22	0,267	-0,49	0,662
	Xanthosine	-1,46	-2,02	-3,60	0,04	-1,55	5	-1,72	0,58	0,02	-1,42	0,14	-2,05	4	-0,83	0,54	-0,58	-1,95	-0,09	-0,33	-0,82	5	-0,76	0,32	0,89	0,312	0,96	0,188
Nucleotides	ADP	0,07	-0,24	-0,27	-0,03	0,17	5	-0,06	0,09	0,28	0,18	-0,19	0,11	4	0,10	0,10	-0,18	-0,23	0,05	0,24	-0,10	5	-0,04	0,09	0,16	0,276	0,02	0,890
	AMP	0,08	0,02	0,04	-0,33	0,30	5	0,02	0,10	-0,06	-0,05	-0,08	0,34	4	0,04	0,10	0,18	0,24	-0,18	0,00	0,09	5	0,06	0,07	0,02	0,912	0,04	0,739
	ATP	-0,63	-0,38	-0,30	-0,74	-0,14	5	-0,44	0,11	0,36	0,18	0,53	0,15	4	0,30	0,09	0,63	0,21	-0,51	-0,15	0,23	5	0,08	0,19	0,74	0,001	0,52	0,046
	CMP	0,09	0,15	-0,37	-0,42	0,11	5	-0,09	0,13	0,09	-0,10	-0,24	0,24	4	0,00	0,10	0,01	0,17	-0,10	-0,03	-0,03	5	0,01	0,04	0,09	0,623	0,10	0,492
	FAD	-0,51	-0,23	-0,27	-0,12	0,17	5	-0,19	0,11	0,15	-0,32	-0,04	0,05	4	-0,04	0,10	0,26	0,21	-0,41	-0,26	0,12	5	-0,02	0,13	0,15	0,355	0,18	0,339
	GDP	-0,41	-0,18	-0,31	0,10	-0,11	5	-0,18	0,09	0,39	0,02	-0,04	0,25	4	0,15	0,10	0,31	0,14	-0,43	-0,43	-0,20	5	-0,12	0,15	0,34	0,040	0,06	0,732
	GMP	0,08	-0,22	-0,42	-0,26	0,02	5	-0,16	0,09	0,03	-0,25	-0,05	0,21	4	-0,02	0,10	0,12	-0,07	-0,16	0,02	-0,04	5	-0,02	0,05	0,15	0,314	0,14	0,222
	NAD	-1,03	-0,50	-0,80	-1,85	-0,93	5	-1,02	0,23	-0,34	-0,64	0,48	-0,16	4	-0,17	0,24	1,35	-0,52	-1,57	-0,61	-0,19	5	-0,31	0,47	0,85	0,036	0,71	0,212
	NADH	-1,33	-0,43	-0,46	-1,19	0,05	5	-0,67	0,26	0,34	-0,31	0,76	0,48	4	0,32	0,23	1,13	0,18	-1,15	-0,41	0,26	5	0,00	0,38	0,99	0,026	0,67	0,181
	NADP	-1,05	-0,31	-0,61	-0,89	-0,41	5	-0,66	0,14	0,75	0,13	0,16	-0,34	4	0,18	0,22	0,28	0	-0,74	0,05	0,11	4	-0,08	0,23	0,83	0,013	0,58	0,058
	NADPH	-1,61	-0,56	0,04	-0,23	-0,36	5	-0,54	0,28	1,26	0,23	0,54	0,59	4	0,66	0,22	0,47	-0,06	-0,90	-0,19	0,47	5	-0,04	0,25	1,20	0,015	0,50	0,223
	NMN	0,00	0,09	0,31	0,04	0,71	5	0,23	0,13	-0,06	-0,12	0,23	0,32	4	0,09	0,11	-0,34	-0,49	-0,54	-0,11	-0,22	5	-0,34	0,08	-0,14	0,456	-0,57	0,006
	UMP	2,72	5,19	5,70	2,54	2,57	5	3,75	0,70	4,96	2,29	2,04	2,44	4	2,93	0,68	2,52	6,14	2,64	5,58	1,27	5	3,63	0,95	-0,81	0,439	-0,12	0,924
Organic acids	2-hydroxybutyric acid	0,06	-0,38	-0,55	-0,12	-0,46	5	-0,29	0,11	-0,77	-1,25	-0,24	0,24	4	-0,51	0,32	0,23	-0,40	-0,54	-0,56	-0,80	5	-0,41	0,17	-0,22	0,510	-0,12	0,564
	2-hydroxyglutaric acid	-0,67	-0,15	-0,25	-0,45	-0,25	5	-0,35	0,09	-0,66	-0,72	-0,18	0,11	4	-0,36	0,20	0,05	0,15	-0,68	-0,46	0,02	5	-0,18	0,16	-0,01	0,972	0,17	0,383
	2-oxoglutaric acid	-0,75	-0,89	-0,20	-1,06	-0,24	5	-0,63	0,17	-1,68	-1,44	-1,07	0,19	4	-1,00	0,42	-0,10	-0,23	-0,91	-1,15	-0,52	5	-0,58	0,20	-0,37	0,398	0,04	0,872
	Allantoin	1,32	3,99	4,03	1,18	1,33	5	2,37	0,67	4,18	1,09	1,18	1,51	4	1,99	0,73	0,83	4,52	1,87	4,36	0,63	5	2,44	0,84	-0,38	0,712	0,07	0,950
	Citric acid;Isocitric acid	-0,38	0,37	-0,63	-0,24	0,44	5	-0,09	0,21	-0,19	-0,44	-0,04	0,08	4	-0,15	0,11	-0,32	-0,03	-0,34	-0,91	-0,21	5	-0,36	0,15	-0,06	0,825	-0,28	0,315
	Fumaric acid	-0,24	-0,70	-0,69	-0,27	0,12	5	-0,35	0,15	-1,01	-0,72	-0,76	0,01	4	-0,62	0,22	-0,07	0,14	-0,30	-0,81	-0,07	5	-0,22	0,16	-0,27	0,336	0,13	0,568
	Hypotaurine	0,27	0,78	1,29	0,70	0,43	5	0,69	0,18	-0,87	-0,75	-1,07	-0,74	4	-0,86	0,08	0,39	0,30	-0,40	0,02	-0,46	5	-0,03	0,17	-1,55	0,000	-0,72	0,019
	Lactic acid	-0,10	-0,21	-0,46	-0,24	-0,15	5	-0,23	0,06	-0,35	-0,45	-0,05	-0,28	4	-0,28	0,08	-0,05	-0,17	-0,15	-0,21	-0,27	5	-0,17	0,04	-0,05	0,623	0,06	0,419
	Malic acid	-0,31	-0,69	-0,73	0,03	0,14	5	-0,31	0,18	-0,76	-0,57	-0,84	-0,06	4	-0,56	0,18	0,01	0,08	-0,23	-0,69	0,25	5	-0,12	0,16	-0,24	0,370	0,20	0,439
	Methylmalonic acid	1,85	5,01	4,50	1,82	2,23	5	3,08	0,69	3,95	1,64	1,40	2,13	4	2,28	0,58	3,28	5,44	1,73	4,88	1,32	5	3,33	0,82	-0,80	0,418	0,25	0,823
	Methylsuccinic acid*	2,28	5,43	5,44	2,40	2,44	5	3,60	0,75	4,55	1,70	1,60	3,36	4	2,80	0,71	3,06	5,58	3,09	5,02	1,10	5	3,57	0,80	-0,79	0,476	-0,03	0,981
	N-acetylputrescine	0,79	-0,58	-0,50	-0,04	0,02	5	-0,06	0,24	-0,69	-0,74	-0,77	-0,26	4	-0,62	0,12	0,10	-0,93	0,39	0,35	-0,46	5	-0,11	0,26	-0,55	0,104	-0,05	0,894
	O-phosphoethanolamine	0,19	-0,46	-0,33	-0,28	0,31	5	-0,12	0,15	-0,87	-0,43	0,23	0,46	4	-0,15	0,30	0,49	-0,14	-0,25	-0,48	-0,74	5	-0,22	0,21	-0,04	0,910	-0,11	0,689
	Phosphoenolpyruvic acid	-0,87	-0,18	-0,49	-1,93	-0,63	5	-0,82	0,30	-0,26	-0,61	-0,83	-0,85	4	-0,64	0,14	0,22	-0,33	-1,30	-0,39	-0,12	5	-0,38	0,25	0,18	0,629	0,43	0,301
	Pyruvic acid;Oxaloacetic acid	0,09	-0,33	-0,52	-0,97	-0,03	5	-0,35	0,19	-0,89	-1,00	-0,26	-0,55	4	-0,67	0,17	0,22	-0,25	-0,18	-0,38	-0,57	5	-0,23	0,13	-0,32	0,255	0,12	0,623
	Succinic acid	-0,64	0,02	-0,72	-0,95	-0,27	5	-0,51	0,17	-1,12	-0,75	-0,80	-0,46	4	-0,78	0,13	0,85	0,03	-1,13	-0,75	-0,21	5	-0,24	0,34	-0,27	0,270	0,27	0,500
	Taurine	-0,87	0,04	-0,31	-0,97	0,40	5	-0,34	0,26	-0,53	-1,14	0,52	2	3	-0,38	0,48	-0,24	1,71	-1,50	-0,92	-0,68	5	-0,32	0,55	-0,04	0,940	0,02	0,976
	Urea	-0,45	-0,57	-0,52	-0,68	0,00	5	-0,44	0,12	-0,95	-1,00	-0,43	-0,06	4	-0,61	0,22	-0,04	0,03	-0,38	-0,81	-0,62	5	-0,36	0,16	-0,16	0,516	0,08	0,695
	SuccinylCoA	-0,52	0,14	-0,74	0,14	-0,05	5	-0,20	0,18	-0,01	-0,83	0,21	1,08	4	0,11	0,39	0,94	0,01	0,46	0,67	-0,03	5	0,41	0,19	0,31	0,459	0,62	0,046

Table S2: Metabolite changes in hepatic cells from control and PD mouse models (GS-PD or ai-PD). N: number of mice/groups detected for each metabolite; Average: Average log2area; SEM: standard error of the mean; Log2FC: Log2 fold change between two groups and statistical analysis was performed by obtaining *p* values (GS-PD vs control, first column Log2FC; ai-PD vs control second column Log2FC). PCae (phosphatidylcholine); PCee (phosphatidylethanolamine).
